# Supplementary material for: The regulatory impact of RNA-binding proteins on microRNA targeting
Source: Nat Commun. 2021 Aug 20;12:5057. doi: 10.1038/s41467-021-25078-5 (PMC8379221; doi:10.1038/s41467-021-25078-5)
Supplement: Supplementary file 1 — Supplementary Information [file 41467_2021_25078_MOESM1_ESM.pdf]

Supplementary Information

The regulatory impact of RNA-binding proteins on microRNA targeting

Sukjun Kim, Soyoung Kim, Hee Ryung Chang, Doyeon Kim, Junehee Park, Narae Son, Joori Park, Minhyuk Yoon, Gwangung Chae, Young-Kook Kim, V. Narry Kim, Yoon Ki Kim, Jin-Wu Nam, Chanseok Shin, Daehyun Baek

Supplementary Discussion 2
Supplementary Figures 6
Supplementary Tables 22
Supplementary Methods 42
References 59

Supplementary Discussion

Robust association between RBP binding and enhanced miRNA targeting efficacy

In our analyses, target mRNAs might unintentionally include a number of false positives because we defined miRNA target sites (MTSs) as canonical 7, 8mer sites. However, when only the MTSs with 8mer site type were examined, which has a significantly reduced false positive rate (**Fig. 6a**), the association between RBP binding and enhanced MT efficacy was consistently observed. To more directly address this issue, we iterated the association analysis on a subgroup of MTSs that are strongly enriched for true positives (context score < -0.1). Likewise, we found that RBP binding was associated with enhanced MT efficacy for a subgroup of MTSs with good context (**Supplementary Fig. 5b**). On the other hand, we mainly utilized RBP-binding sites (RBSs) provided by ENCODE eCLIP-seq in our analyses, which can also include false positives due to the limitation of technology. However, the association was consistently observed for more reliable RBSs supported by binding motifs (**Supplementary Fig. 2e**). From these consistent results, although some false positives would be included among the MTSs and RBSs used in the investigation, we believe that the association we observed is robust and valid.

Independent measurement of the distance between an MTS and the nearest RBS

The most ideal way of estimating the distance between an MTS and the nearest RBS on the 3′UTR, denoted as $d_{\text{MTS-RBS}}$, is to measure it only once for each MTS by combining RBSs from all human RBPs. However, it is impossible at the current stage because an exhaustive list of all RBSs in human has not yet been characterized and because the current CLIP technology has the limitation that it can capture the interactions for one RBP at a time. Besides, there has been no experimental method to determine a complete picture of protein-RNA interactions for all RBPs at once. Therefore, one of the most feasible ways is to define the distance from an MTS to the nearest RBS for each RBP as $d_{\text{MTS-RBS}}$, and regard this observation as an independent measurement for the MTS and this is how we made observations. It is also appropriate and common method for analyzing eCLIP-seq datasets, that focuses on a single RBP without considering other RBPs.

The association of $d_{MTS\text{-}RBS}$ measured as above with MT efficacy could be confounded by 3′UTR length, which is known to determine miRNA targeting efficacy. To rule out this confounding effect, we carefully selected a group of 3′UTRs that have different $d_{MTS\text{-}RBS}$ but have statistically indistinguishable confounding factors as explained in **Methods**. Our observation was still consistent even after a rigorous correction of the potential confounding effects including the 3′UTR length (**Fig. 1d**), supporting that the association between $d_{MTS\text{-}RBS}$ and MT efficacy is not biased by analytical artifacts.

Mechanistic view on how RBP influences RNA structure

As discussed in main text, a large number of previous studies have provided multiple lines of evidence supporting the ability of RBPs to mediate the unfolding of RNA secondary structures, including transcriptome-wide RNA structure probing[1] and experimental validation on individual RBP[2]. RBPs with low binding affinity can open RNA structure via both active and passive mechanisms. Active mechanism corresponds to the function of ATP-dependent helicases, which take a small portion of RBPs. In addition to the helicases, a number of RBPs have been revealed to function as RNA destabilizing chaperone, or to capture the transient, less stable state of RNAs[3, 4]. Once RNA is destabilized by either active or passive mechanism, the state presumably is maintained by binding of additional RBPs. The consistent associations between RBP binding and enhanced MT efficacy regardless of the helicase activity also give us a clue that both active and passive mechanisms are involved in the unfolding of RNA secondary structure (**Fig. 6d**).

In order to more directly examine whether RBP binding can open RNA structure, we compared DMS scores between *in vivo* and *in vitro.* As a result, the DMS scores for 3′UTR fragments containing RBSs were increased *in vivo* condition compared to *in vitro*, and the level of increment was higher than that of 3′UTR fragments without any RBP binding ('No RBS' group) even after careful control of confounding factors (**Fig. 4b**). The increased *in vivo* DMS score was more pronounced when a 3′UTR fragment contains stringent RBSs or a larger number of RBPs bind to the fragment (**Fig. 4c**). When examining individual RBPs, the increased *in vivo* DMS score was detected and the levels of increase were higher than 'No RBS' group for most of RBPs (119 of 120 RBPs, **Fig. 4d**). These results provide us evidence that the RBP binding can induce the opening of the structured RNAs, and this opening can be mediated by almost all RBPs.

Our result of **Fig. 4d** indicates the binding of double-stranded RNA-binding proteins (dsRBPs) as well as single-stranded RNA-binding proteins is associated with opening of RNA secondary structure. In addition, consistent associations between $d_{\text{MTS-RBS}}$ and MT efficacy were observed for subsets of RBPs separated by strand specificity, indicating that the binding of RBPs enhances MT efficacy regardless of their strand specificity (**Fig. 6e**). The mechanism of how the binding of dsRBPs enhances MT efficacy is probably through their dual capability of binding both ssRNAs and dsRNAs. For instance, it is known that DGCR8, one of the dsRBPs that we analyzed, binds ssRNAs and dsRNAs with similar affinities[5].

Double-stranded RBPs (dsRBPs) and nuclear RBPs

We have observed that dsRBPs can also influence MT efficacy even when carefully selecting a subset of dsRBPs with minimal overlapping binding sites with those of ssRBPs (**Supplementary Fig. 6c**). However, our result should be cautiously interpreted because it could still be confounded by overlapping sites of ssRBPs that have not examined yet. Moreover, UV crosslinking bias to single-stranded regions makes it difficult to investigate the impact of dsRBP binding to double-stranded regions on MT efficacy.

Also for the RBPs enriched in the nucleus rather than the cytoplasm, significant association of $d_{\text{MTS-RBS}}$ with MT efficacy, comparable to that of RBPs with high cytoplasmic fraction, were observed. It indicates that even binding of RBPs with low cytoplasmic fraction appears to induce the enhanced MT efficacy (**Fig. 6f and Supplementary Fig. 6d**). Similarly to the problems associated with our results of dsRBPs, this result may be confounded by overlapping binding sites with those of the cytoplasmic RBPs. Unfortunately, it is infeasible to completely eliminate RBPs that have overlapping binding sites with currently available data. More accurate analysis on dsRBPs and nuclear RBPs would be possible when more comprehensive eCLIP-seq datasets and unbiased methods to capture RBSs become available in the future.

Motif-specific *vs.* motif-unspecific RBP-binding sites

The binding affinity of RBSs would affect the occupancy of RBSs, raising a possibility that it will also affect the regulatory impact of RBPs on MT. Regarding this issue, we have utilized *in vivo* RBP-binding motif database mCross[6] in our analysis, to investigate association between the number of RBPs bound close to the MTS and MT efficacy with motif-specific RBSs which are expected to have high affinity (**Supplementary**

Fig. 2e). According to our results, enrichment of motif-specific RBSs was clearly associated with enhanced MT, and a similar pattern was observed for motif-unspecific RBSs as well. We also performed the analysis of **Fig. 1d** with motif-specific RBSs and observed consistent association between $d_{\text{MTS-RBS}}$ and MT efficacy (**Supplementary Fig. 5b**), supporting the robustness of our findings. Taking these results, although the occupancy of some RBSs can be relatively low due to the motif-specificity or intracellular localization of the RBPs, it is sufficient enough to enhance MT efficacy on a global scale.

Supplementary Figures

Supplementary Figure 1

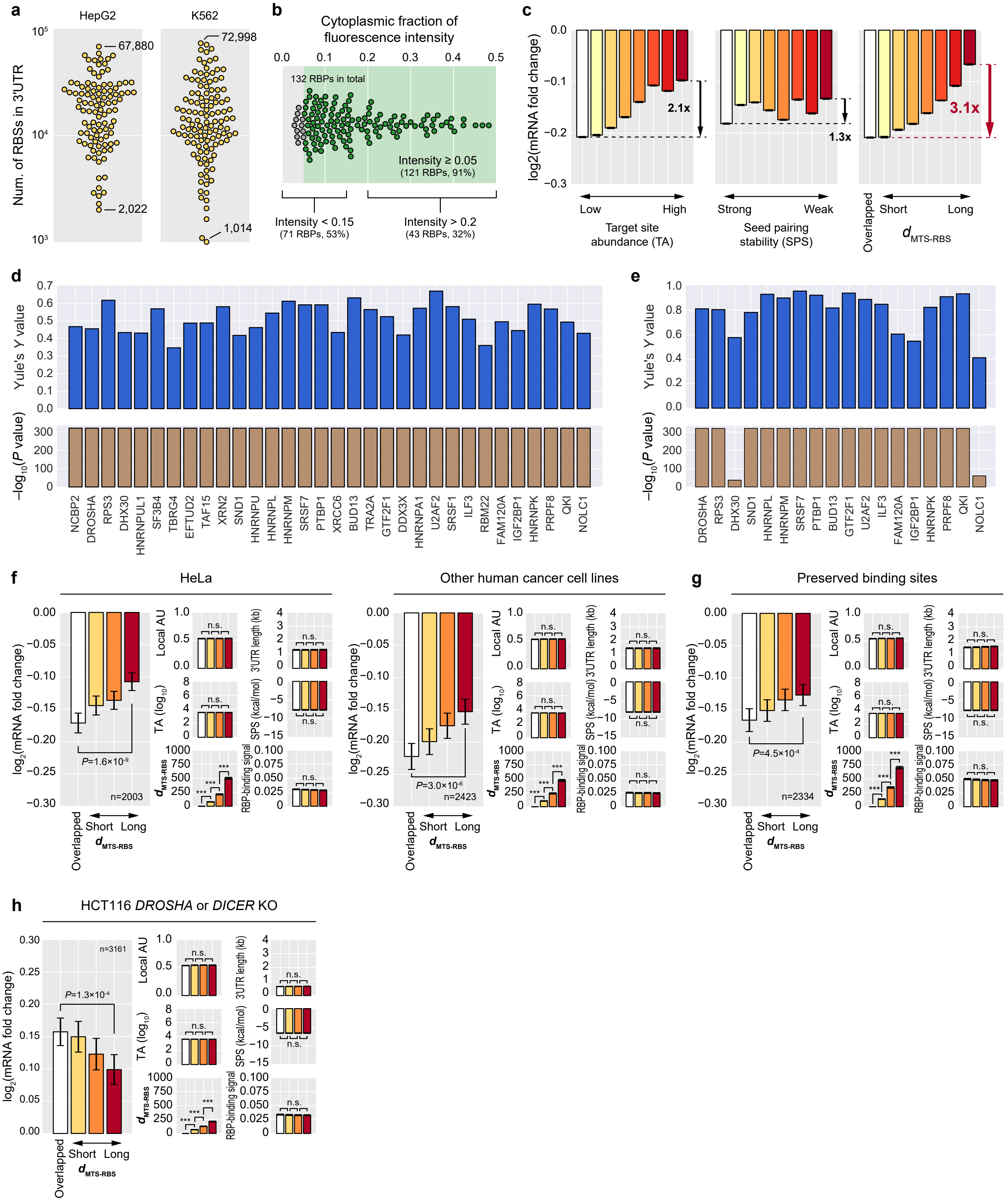

Supplementary Figure 1. Analysis of basic properties of RBPs and their effect on miRNA targeting efficacy.

a, The number of 3′UTR binding sites for each RBP is shown on a $\log_{10}$ scale. The ENCODE eCLIP-seq RBP-binding sites (RBSs) located in 3′UTRs have been enumerated by analyzing the binding information profiled in K562 (left) and HepG2 (right) cell lines.

b, The cytoplasmic fraction of RBPs. The fluorescence intensities of individual RBPs were measured by fluorescence *in situ* hybridization (FISH) images in HepG2 and HeLa cell lines. The cytoplasmic fraction compared to the nuclear fraction for each RBP is shown. The RBPs with cytoplasmic fraction >0.2 and <0.15 were used in the analysis shown in **Fig. 6f**.

c, Comparison of miRNA targeting efficacies affected by various determinants. From the datasets of HepG2, HeLa, and other human cancer cell lines, target genes with a single 7, 8mer site for corresponding miRNA were collected. For each equal-sized bin separated by target site abundance (TA; left), seed pairing stability (SPS; middle), and the distance between a miRNA target site and the nearest RBS ($d_{\text{MTS-RBS}}$; right), the mean $\log_2$(mRNA fold change) was plotted. The error bars represent 95% confidence intervals. The number of 3′UTRs for each bin are provided in Source Data.

d, **e**, Robust preservation of RBP binding between the different cell lines. Yule's *Y* value was used to calculate the co-occurrence of 3′UTR RBSs between HepG2 and K562 cell lines (top), and the statistical significance was computed using the χ^2 test for a 2×2 contingency table (bottom). Leniently and stringently called RBSs were used in **d** and **e**, respectively.

f, Full versions of **Fig. 1d** including the information for the correction of the potentially confounding factors are shown. Otherwise as in **Fig. 1d**.

g, Association analysis between the $d_{\text{MTS-RBS}}$ and MT efficacy using the preserved RBSs. Otherwise as in **Fig. 1d**.

h, Full versions of **Fig. 1e** including the information for the correction of the potentially confounding factors are shown. Otherwise as in **Fig. 1d**.

Supplementary Figure 2

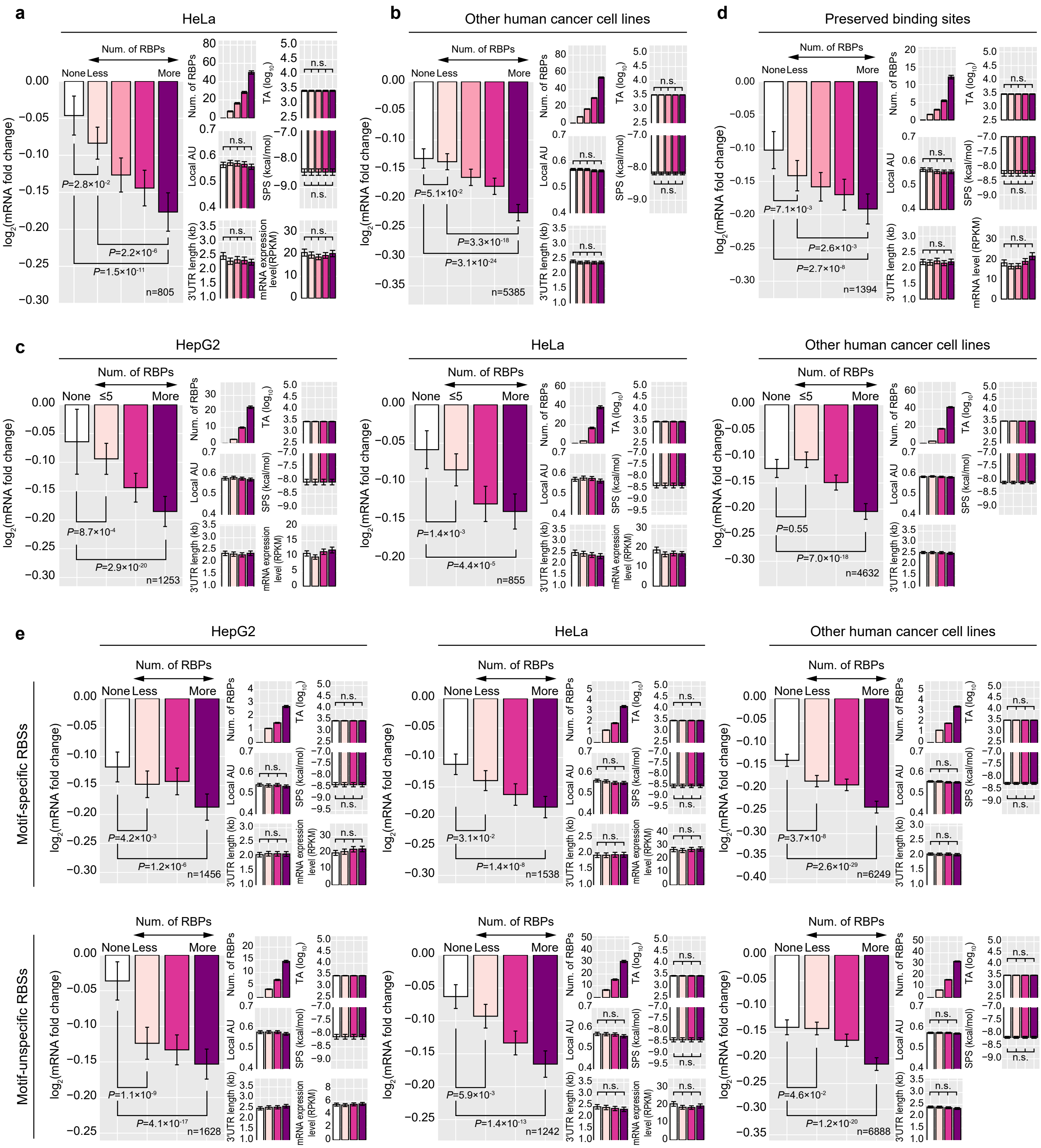

Supplementary Figure 2. Association analysis between the number of RBPs bound close to the miRNA target site (MTS) and miRNA targeting (MT) efficacy.

a, **b**, Full version of **Fig. 3a** for HeLa and other human cancer cell lines are shown including the information for the correction of the potentially confounding factors. For HeLa (**a**) and other human cancer cell lines (**b**) datasets, 3′UTRs were separated into five subgroups with respect to the number of RBPs bound within 50 nts from the miRNA target site. Otherwise as in **Fig. 3a**.

c, Association analyses between a small number of bound RBPs close to the MTS and MT efficacy. 3′UTRs were separated into four subgroups with respect to the number of RBPs bound within 50 nts from the MTS. To specifically examine the impact of a small number of bound RBPs on MT, in the second subgroup, we chose those MTSs that include five or less number of bound RBPs. Each subgroup was carefully selected to have statistically indistinguishable confounding features among subgroups (see Methods). mRNA fold changes and values of confounding features were compared among these subgroups (two-sided Wilcoxon's rank-sum test) with the mean values of confounding features and $\log_2$(mRNA fold change) displayed. The association analysis shown in **Fig. 3a** was performed for HepG2 (left), HeLa (middle), and other human cancer cell lines (right). Otherwise as in **Fig. 3a**.

d, Association analysis between the number of RBPs bound close to the MTS and MT efficacy using preserved RBSs between two cell lines (K562 and HepG2). Otherwise as in **Fig. 3a**.

e, Association analyses between the number of RBPs bound close to the MTS and MT efficacy with respect to the motif specificity. For each of human cell lines (HepG2, HeLa, and other human cancer cell lines), RBP-binding sites were divided into motif-specific (top) and motif-unspecific RBP-binding sites (bottom) depending on whether or not they contain sequence motifs of each RBP, respectively. Otherwise as in **d**.

Supplementary Figure 3

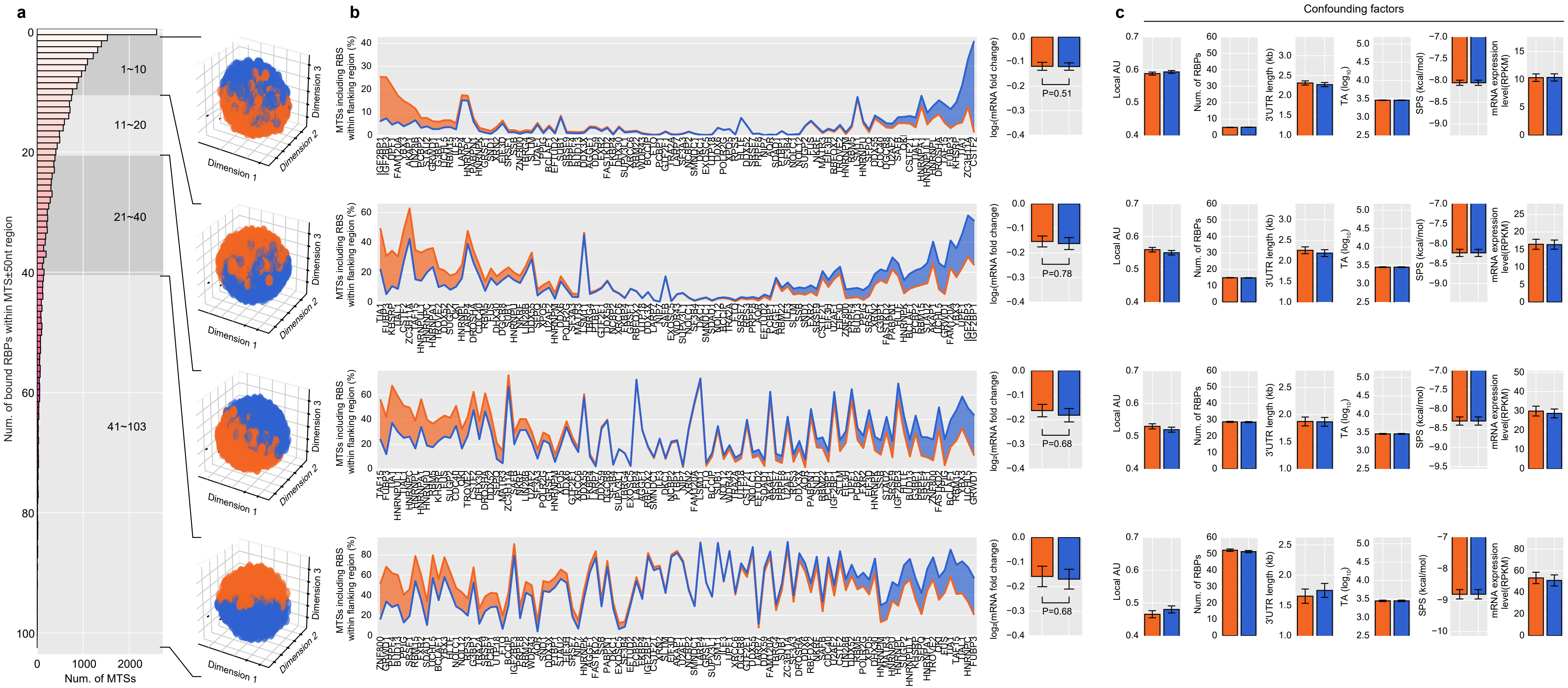

Supplementary Figure 3. The overall number of bound RBPs instead of specific identities of RBPs determines miRNA targeting (MT) efficacy in HepG2 cell line.

a-**c**, Full version of **Fig. 3b, c**. Distribution of miRNA target sites (MTSs) with respect to the number of RBPs bound within 50 nt flanking regions in HepG2 data and the separation of MTSs by the composition of bound RBPs (**a**). The RBP compositions and the average MT efficacies are depicted and compared (two-sided Wilcoxon's rank-sum test) (**b**), after the correction of the potentially confounding factors (**c**) as following. The pairs of MTSs from the two subgroups with similar values of the known confounding features (local AU content, target abundance, seed pairing stability, and 3′UTR length), mRNA expression level (RPKM), and the number of RBPs bound within 50 nt flanking regions were sampled. The sampling process was repeated for the MTSs whose number of RBPs bound within 50 nt flanking regions are from 1 to 10, from 11 to 20, from 21 to 40, and from 41 to 78. The values of each feature between the sampled MTS subgroups were compared (two-sided Wilcoxon's rank-sum test), and no statistically significant difference was found in any of the comparisons (P>0.05). The mean values of confounding factors and $\log_2$(mRNA fold change) are displayed, and the error bars represent 95% confidence intervals. The number of examined 3′UTRs are provided in Source Data. Otherwise as in **Fig. 3b, c**.

Supplementary Figure 4

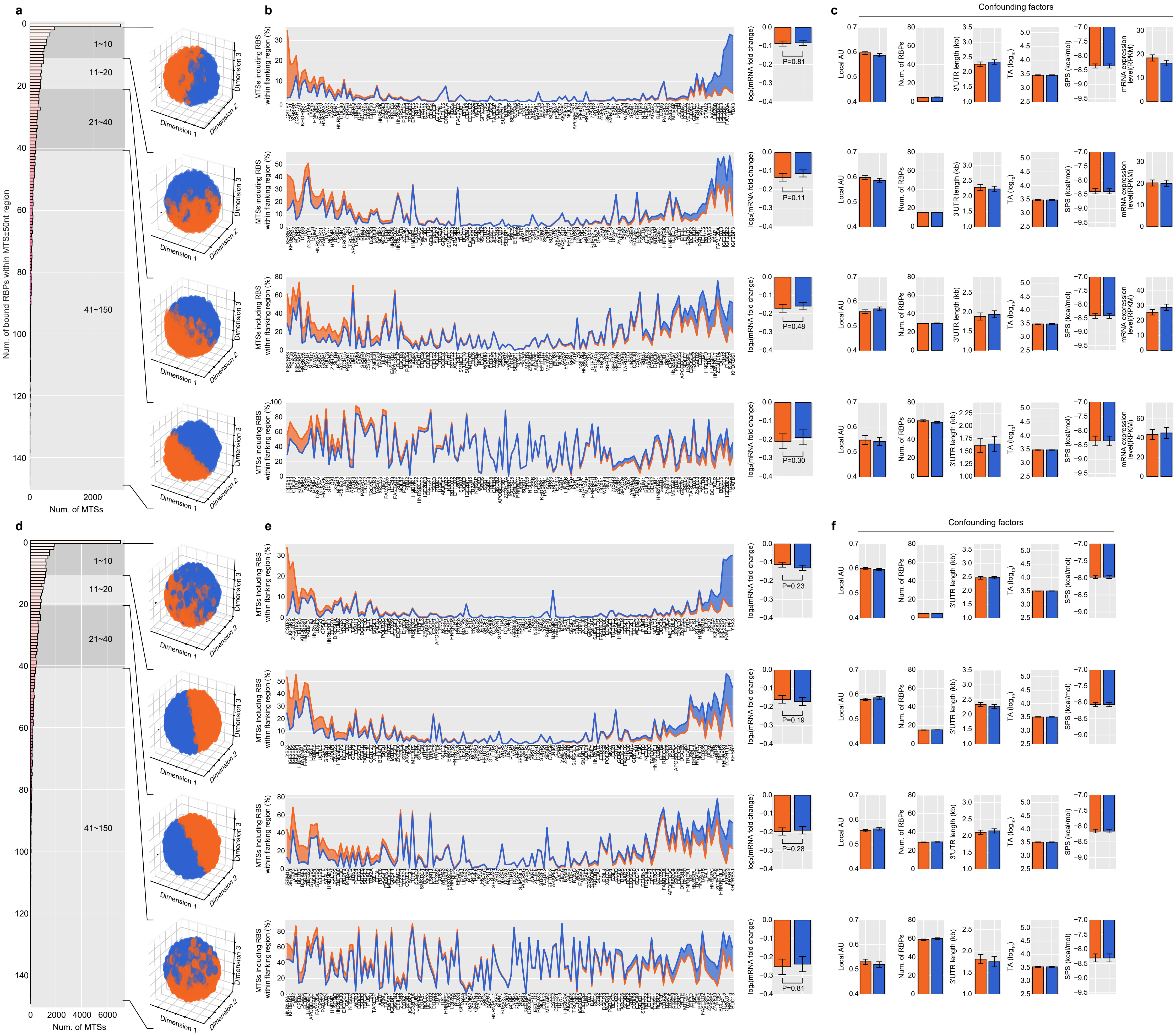

Supplementary Figure 4. The overall number of bound RBPs instead of specific identities of RBPs determines miRNA targeting (MT) efficacy in HeLa and other human cancer cell lines.

a-**f**, Distribution of miRNA target sites (MTSs) with respect to the number of RBPs bound within 50 nt flanking regions in HeLa (**a**) and other human cancer cell lines (**d**) datasets and the separation of MTSs by the composition of bound RBPs in HeLa (**a**) and other human cancer cell lines (**d**). The RBP compositions and the mean MT efficacies are depicted and compared (**b**, **e**) after the correction of the potentially confounding factors (**c**, **f**). In both datasets, binding information of all available 150 RBPs profiled in HepG2 and K562 cell lines was used for the analysis. Otherwise as in **Supplementary Fig. 3**.

Supplementary Figure 5

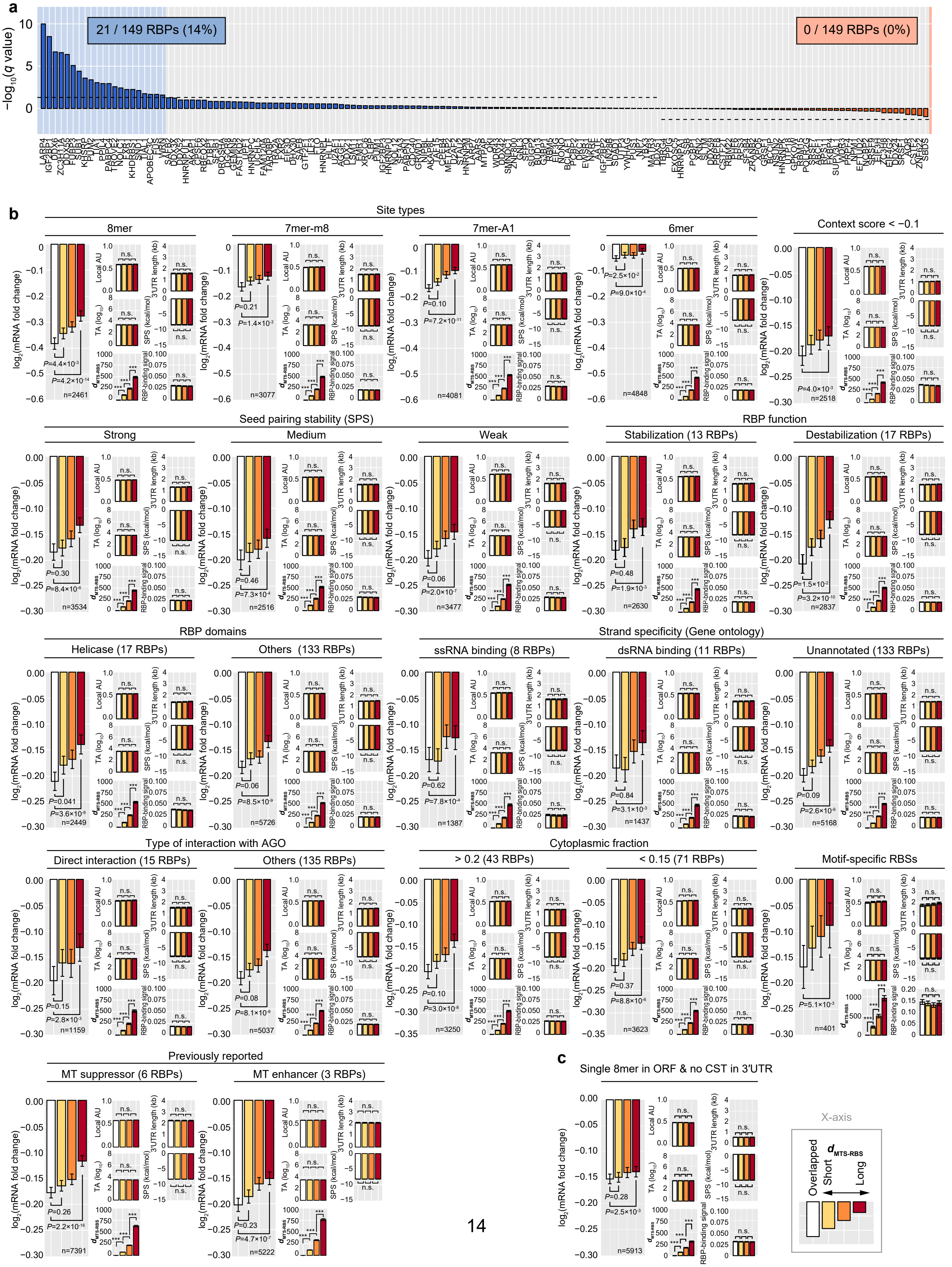

Supplementary Figure 5. Analyses on a potential mechanism by which RBP binding enhances miRNA targeting.

a, Comparison of the ternary interaction model and our proposed model for individual RBPs. For each RBP, the ternary interaction model and our proposed model of miRNA targeting were constructed for multiple linear regression. Using one-sided Steiger's *z* test, the two models were compared to examine whether which of the two models better fits to the observed data. *P* values obtained from the test were corrected by the false discovery rate, and the *q* values for each RBP are shown in $\log_{10}$ scale. Upward and downward directions of the y-axis indicate whether our proposed model explains miRNA targeting efficacy better or worse than the ternary interaction model, respectively. The horizontal dashed lines indicate the *q* value cutoff of 0.01.

b, Full versions of **Figs. 4a** and **6a-g** including the information for the correction of the potentially confounding factors. Otherwise as in **Figs. 4a** and **6a-g**.

c, The impact of RBP binding on miRNA targeting in ORF. The genes with a single 8mer miRNA target site (MTS) in ORF and without any 6-8mer target site in 3′UTR were selected and split into four groups with respect to distance between the MTS and the RBP-binding sites measured on ORF. mRNA fold changes measured from HepG2, HeLa, and other human cancer cell lines were compared after each subgroup was carefully chosen to have statistically indistinguishable features such as the four known confounding features and average RBP-binding signal of the nearest RBP-binding site, among four subgroups. Otherwise as in **Fig. 1d**.

Supplementary Figure 6

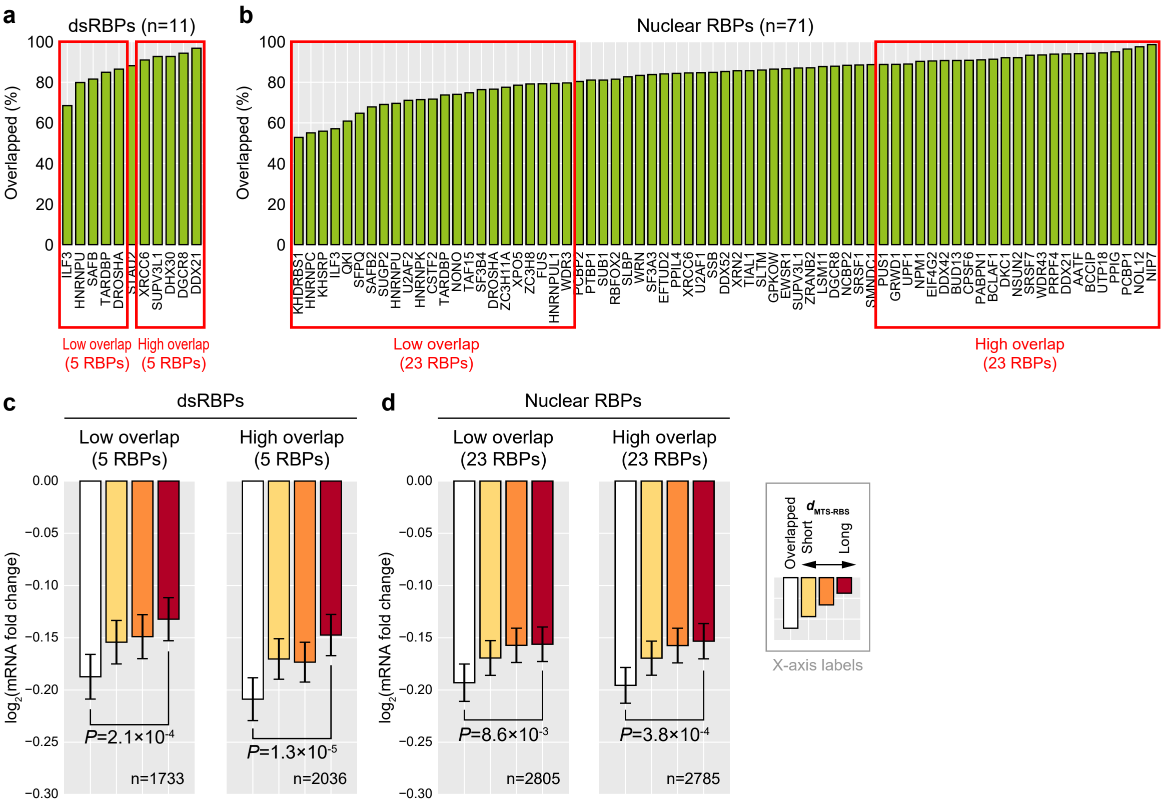

Supplementary Figure 6. Association analysis between MT efficacy and binding of double-stranded RNA binding proteins (dsRBPs) or nuclear RBPs.

a, b, Relative fraction of the RBP-binding sites (RBSs) of dsRBPs (**a**) or nuclear RBPs (**b**) overlapped with RBSs of other RBPs. For more details, see **Supplementary Methods**.

c, d, Association analysis with dsRBPs (**c**) or nuclear RBPs (**d**) with low or high fraction of overlap with other RBPs. Otherwise as in **Fig. 1d**.

Supplementary Figure 7

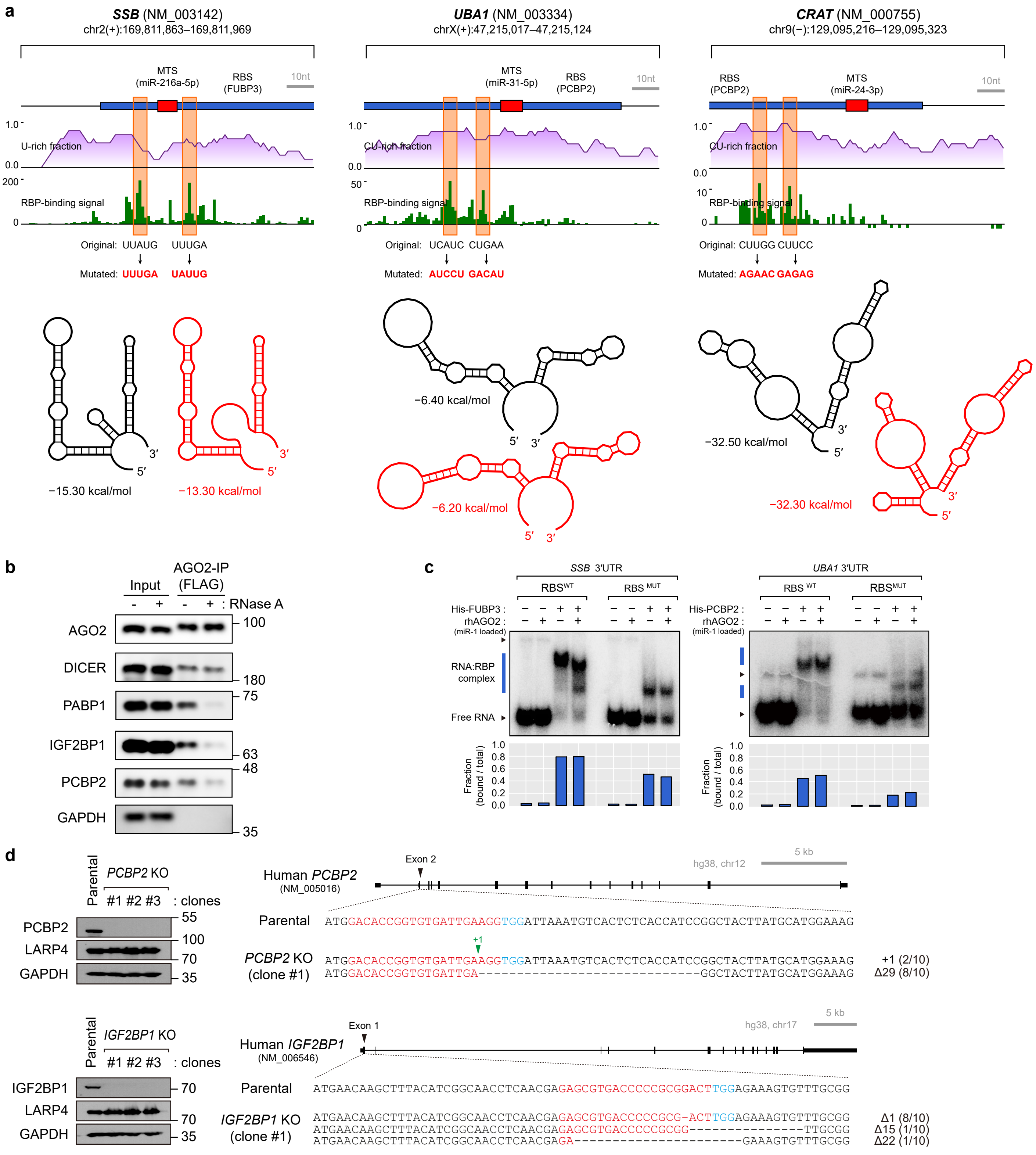

Supplementary Figure 7. RNA constructs and RBP knockout cell lines for experimental validations.

a, Illustration of RBP-binding profile of RNA constructs used in gel mobility-shift assay. For gel mobility-shift assay, the 50 nt flanking regions from the selected miRNA target sites (indicated as a red box) were used to build an RNA construct. RBP-binding sites (indicated as blue boxes), U- or CU-rich fraction (line graph), and RBP-binding signals (green bars) for the corresponding region are shown. Mutated sites selected from the two strongest peaks across RBP-binding signals are represented as orange boxes. The chromosomal position of the depicted region is displayed on top.

b, Co-immunoprecipitation of PCBP2 and IGF2BP1 after RNase A treatment. Flag-AGO2 overexpressed HEK293T cells were lysed, treated either with RNase A or without, and then immunoprecipitated. The co-immunoprecipitated DICER, PABP1, IGF2BP1, PCBP2, and GAPDH were detected by western blotting. The blot is representative of two replicates. DICER and PABP1, known AGO2 interactor and non-interactor, respectively, were used as controls.

c, Gel mobility-shift assays for RBS^{WT} and RBS^{MUT} of 3′UTRs with RBPs and rhAGO2 loaded with a non-targeting miRNA. Gel mobility-shift assays were performed in 4 different conditions: (1) with 3′UTR only, (2) with 3′UTR and rhAGO2, (3) with 3′UTR and RBP, and (4) with 3′UTR, RBP, and rhAGO2. The results with a non-targeting miRNA (miR-1) are shown. Otherwise as in **Fig. 7c, d**.

d, Generation of RBP knockout cell lines using CRISPR-Cas9 system. Validation of the disruption of *PCBP2* (top) and *IGF2BP1* (bottom) in HEK293T cells by western blot analysis. Both LARP4 and GAPDH protein levels are shown as loading controls (left). The blot is representative of two replicates. PCR-based genotypes of each representative RBP KO clone are aligned to their parental sequences (right). The guide RNA target sites for CRISPR-Cas9 are highlighted in red and their PAM sequences are in blue. The indels of 10 PCR clones were sequenced by the Sanger sequencing and are indicated by hyphens.

Supplementary Figure 8

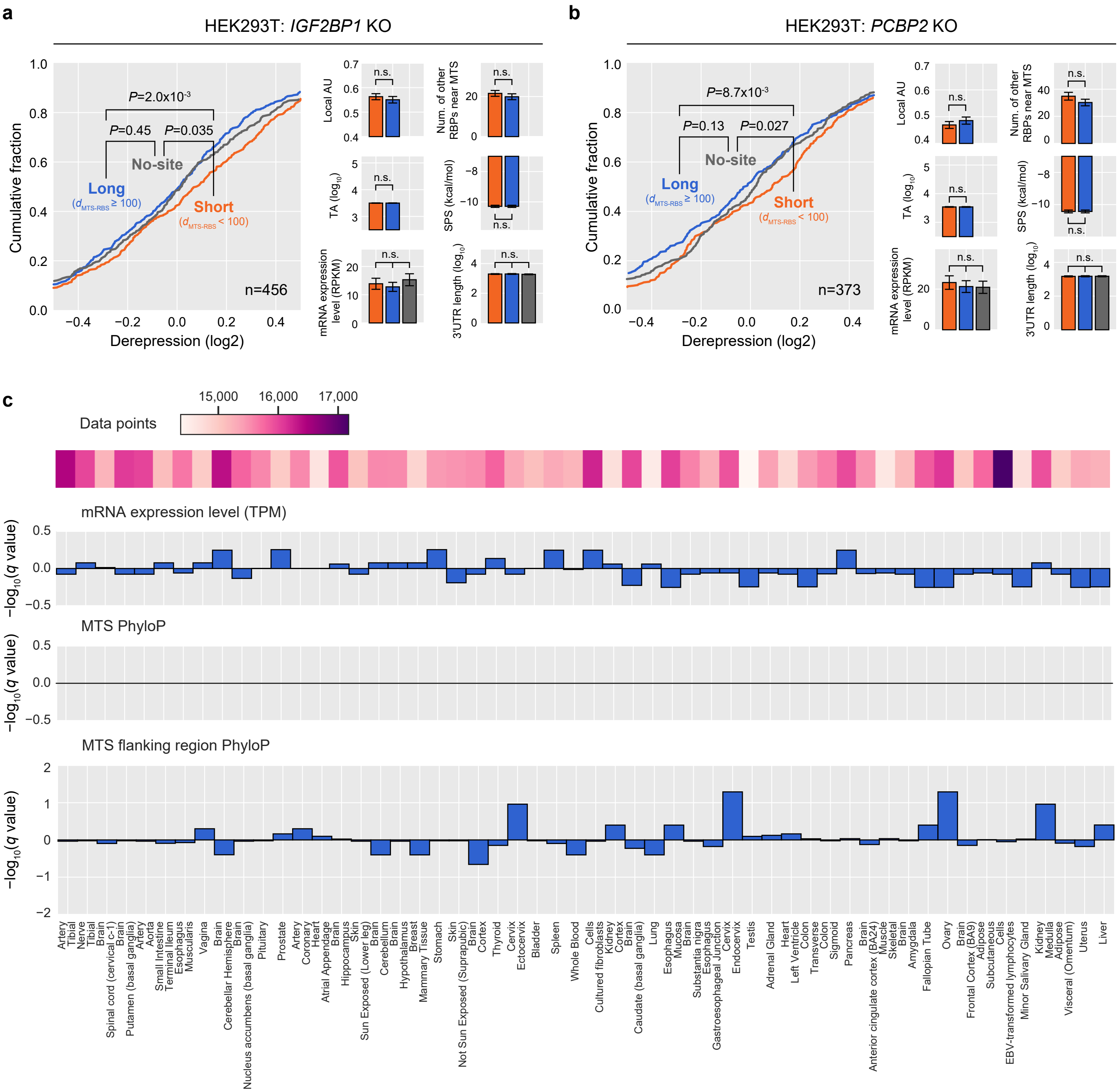

Supplementary Figure 8. Transcriptome-wide response and evolutionary perspective on the regulatory impact of RBPs on miRNA targeting.

a, **b**, Transcriptome-wide response of miRNA targets after RBP removal for individual RBPs. The analysis shown in **Fig. 8c** was repeated for mRNA-seq data after KO of individual RBPs, *PCBP2* (**a**) or *IGF2BP1* (**b**). Otherwise as in **Fig. 8c**.

c, Correction for confounding features when evaluating co-occurrence of evolutionarily conserved miRNA target sites (MTSs) and RBP-binding sites. For each of 54 human tissues, the MTSs of conserved miRNAs with high expression levels and the MTSs of the control miRNAs in corresponding tissue were collected. To prevent bias by potentially confounding factors, pairs of MTSs for conserved miRNAs and their controls were selected to have statistically indistinguishable difference of mRNA expression level (second row), evolutionary conservation of MTS (third row), and conservation of flanking region around MTS (fourth row). The number of selected pairs for each tissue are shown on top. Otherwise as in **Fig. 9b**.

Supplementary Tables

Supplementary Table 1. Previously reported binding affinities of RBPs and miRNA-loaded AGO

Dissociation constant (K_D) values of RBPs collected through literature survey are listed. The relative fold value compared to the K_D between let-7 loaded AGO and an 8mer miRNA target site is shown for each RBP.

No.	Name of RBP or AGO	K_D	Fold	Reference
1	SAM68	$1.2x10^{-8}$	800.0	Lin *et al.*, 1997, JBC
2	U1A, Fox-1	$5.0x10^{-10}$	33.3	Chen & Varani, 2013
3	MBNL1	$3.1x10^{-9}$	206.7	Jahromi *et al.*, 2013
4	HuR	$2.5x10^{-9}$	166.7	D'Agostino *et al.*, 2013
5	PUM1	$4.8x10^{-10}$	32.0	Cheong & Hall, 2006
6	TFIIIA (binding to 5s rRNA)	$1.0x10^{-10}$	6.7	Friesen & Darby, 1997
7	ZRANB2	$2.0x10^{-7}$	13,333.3	Loughlin *et al.*, 2009
8	TTP73	$1.9x10^{-8}$	1,266.7	Blackshear *et al.*, 2003
9	TIA1	$1.7x10^{-8}$	1,133.3	Wang *et al.*, 2014
10	PTBP, SRSF3	$1.0x10^{-8}$	666.7	Helder *et al.*, 2016
11	MEX-3C	$1.2x10^{-7}$	8,000.0	Yang *et al.*, 2017
12	IRE-IRP1	$1.4x10^{-11}$	0.9	Philpott *et al.*, 1994
13	IRE-IRP1 (Fe^{2+} depleted)	$2.0x10^{-11}$	1.3	Haile *et al.*, 1989
14	IRE-IRP1 (Fe^{2+} abundant)	$3.0x10^{-9}$	200.0	Haile *et al.*, 1989
15	MS2 binding protein	$3.0x10^{-9}$	200.0	Lim *et al.*, 1994
16	HNRNPA1	$1.0x10^{-9}$	66.7	Burd *et al.*, 1994
17	Galectin-3	$2.3x10^{-6}$	153,333.3	Wang *et al.*, 1995
18	FMR1	$5.7x10^{-9}$	380.0	Ashley *et al.*, 1993
19	HNRNPC	$1.0x10^{-8}$	666.7	Gorlach *et al.*, 1994
20	Tristetraprolin (ZFP36)	$3.6x10^{-9}$	240.0	Brewer *et al.*, 2004
21	TDP43 (TARDBP)	$3.5x10^{-10}$	23.3	Flores *et al.*, 2019
22	TDP43 (TARDBP)	$2.26x10^{-8}$	1,506.7	Lukavsky *et al.*, 2013
23	GEMIN5	$2.4x10^{-7}$	16,000.0	Jin *et al.*, 2016
24	HNRNPL	$8.5x10^{-9}$	566.7	Yao *et al.*, 2017
25	Mouse hepatitis virus N protein	$1.4x10^{-8}$	933.3	Nelson *et al.*, 2000
26	IGF2BP1	$4.4x10^{-8}$	2,933.3	Huang *et al.*, 2018

27	IGF2BP2	$1.6x10^{-8}$	1,066.7	Huang *et al*., 2018
28	IGF2BP3	$3.15x10^{-7}$	21,000.0	Huang *et al*., 2018
29	IGF2BP2	$3.0x10^{-8}$	2,000.0	Hafner *et al*., 2010
30	QKI	$9.3x10^{-8}$	6,200.0	Hafner *et al*., 2010
31	EIF4B/4F+PABP	$1.8x10^{-8}$	1,200.0	Yumak *et al*., 2010
32	U2AF2	$3.2x10^{-7}$	21,333.3	Sutandy *et al*., 2018
33	RBFOX2	$5.0x10^{-9}$	333.3	Lambert *et al*., 2014
34	PCBP1	$2.4x10^{-6}$	160,000.0	Evans *et al*., 2003
35	PCBP2	$1.6x10^{-5}$	1,066,666.7	Evans *et al*., 2003
36	HNRNPK	$7.8x10^{-6}$	520,000.0	Evans *et al*., 2003
37	let-7 loaded AGO (mouse), 7mer-m8 site	$2.6x10^{-11}$	1.7	Wee *et al*., 2012
38	let-7 loaded AGO (mouse), 8mer site	$1.5x10^{-11}$	1.0	Salomon *et al*., 2015

Supplementary Table 2. NGS samples and microarrays used in the study

List of high-throughput sequencing samples and microarrays are shown with metadata information. (1) Accession: GEO accession identifier for the individual samples, (2) Cell: the name of the cell lines, (3) Condition: parental cell line or specific protein knockout, (4) Assay: experiment type of NGS. 'small RNA-seq' for sequencing of small RNA libraries, 'mRNA-seq' for sequencing of mRNA libraries and 'DMS-seq' for sequencing of DMS-untreated and DMS-treated libraries, (5) Treatment: name of miRNAs transfected to the cell (for mRNA-seq) or whether the DMS is treated (for DMS-seq), (6) Conc.: the concentration of transfected miRNA (unit: nM), (7) Incub.: incubation time after transfection (unit: hours).

NGS samples

No.	Accession	Cell	Condition	Assay	Treatment	Conc.	Incub.
1	GSM3788539	HEK293T	-	small RNA-seq	-	-	-
2	GSM3788540	HeLa	-	small RNA-seq	-	-	-
3	GSM3788541	HCT116	-	small RNA-seq	-	-	-
4	GSM3788542	HepG2	-	small RNA-seq	-	-	-
5	GSM3788543	HeLa	-	mRNA-seq	-	-	-
6	GSM3788544	HEK293T	-	mRNA-seq	-	-	-
7	GSM3788545	HCT116	-	mRNA-seq	-	-	-
8	GSM3788546	HepG2	-	mRNA-seq	-	-	-
9	GSM3788547	HepG2	-	mRNA-seq	mock	-	48
10	GSM3788548	HepG2	-	mRNA-seq	mock	-	48
11	GSM3788549	HepG2	-	mRNA-seq	mock	-	48
12	GSM3788550	HepG2	-	mRNA-seq	mock	-	48
13	GSM3788551	HepG2	-	mRNA-seq	mock	-	48
14	GSM3788552	HepG2	-	mRNA-seq	mock	-	48
15	GSM3788553	HepG2	-	mRNA-seq	mock	-	48
16	GSM3788554	HepG2	-	mRNA-seq	mock	-	48
17	GSM3788555	HepG2	-	mRNA-seq	mock	-	48
18	GSM3788556	HepG2	-	mRNA-seq	mock	-	48
19	GSM3788557	HepG2	-	mRNA-seq	mock	-	48
20	-	HepG2	-	mRNA-seq	mock	-	48

21	-	HepG2	-	mRNA-seq	mock	-	48
22	-	HepG2	-	mRNA-seq	mock	-	48
23	-	HepG2	-	mRNA-seq	mock	-	48
24	-	HepG2	-	mRNA-seq	mock	-	48
25	-	HepG2	-	mRNA-seq	mock	-	48
26	GSM3788558	HepG2	-	mRNA-seq	hsa-miR-211-5p	100	48
27	GSM3788559	HepG2	-	mRNA-seq	hsa-miR-211-5p	300	48
28	GSM3788560	HepG2	-	mRNA-seq	hsa-miR-211-5p	200	48
29	GSM3788561	HepG2	-	mRNA-seq	hsa-miR-211-5p	200	48
30	GSM3788562	HepG2	-	mRNA-seq	hsa-miR-18a-5p	100	48
31	GSM3788563	HepG2	-	mRNA-seq	hsa-miR-18a-5p	300	48
32	GSM3788564	HepG2	-	mRNA-seq	hsa-miR-18a-5p	300	48
33	GSM3788565	HepG2	-	mRNA-seq	hsa-miR-18a-5p	200	48
34	GSM3788566	HepG2	-	mRNA-seq	hsa-miR-18a-5p	200	48
35	GSM3788567	HepG2	-	mRNA-seq	hsa-miR-137	100	48
36	GSM3788568	HepG2	-	mRNA-seq	hsa-miR-137	300	48
37	GSM3788569	HepG2	-	mRNA-seq	hsa-miR-137	300	48
38	GSM3788570	HepG2	-	mRNA-seq	hsa-miR-137	200	48
39	GSM3788571	HepG2	-	mRNA-seq	hsa-miR-137	200	48
40	GSM3788572	HepG2	-	mRNA-seq	hsa-miR-140-5p	100	48
41	GSM3788573	HepG2	-	mRNA-seq	hsa-miR-140-5p	300	48
42	GSM3788574	HepG2	-	mRNA-seq	hsa-miR-140-5p	300	48
43	GSM3788575	HepG2	-	mRNA-seq	hsa-miR-133a-3p	100	48
44	GSM3788576	HepG2	-	mRNA-seq	hsa-miR-133a-3p	300	48
45	GSM3788577	HepG2	-	mRNA-seq	hsa-miR-133a-3p	200	48
46	GSM3788578	HepG2	-	mRNA-seq	hsa-miR-133a-3p	200	48
47	GSM3788579	HepG2	-	mRNA-seq	hsa-miR-101-3p	100	48
48	GSM3788580	HepG2	-	mRNA-seq	hsa-miR-101-3p	300	48
49	GSM3788581	HepG2	-	mRNA-seq	hsa-miR-101-3p	200	48
50	GSM3788582	HepG2	-	mRNA-seq	hsa-miR-101-3p	200	48
51	GSM3788583	HepG2	-	mRNA-seq	hsa-miR-140-3p	100	48
52	GSM3788584	HepG2	-	mRNA-seq	hsa-miR-140-3p	300	48
53	GSM3788585	HepG2	-	mRNA-seq	hsa-miR-140-3p	300	48
54	GSM3788586	HepG2	-	mRNA-seq	hsa-miR-199b-3p	100	48

55	GSM3788587	HepG2	-	mRNA-seq	hsa-miR-199b-3p	300	48
56	GSM3788588	HepG2	-	mRNA-seq	hsa-miR-199b-3p	200	48
57	GSM3788589	HepG2	-	mRNA-seq	hsa-miR-199b-3p	200	48
58	GSM3788590	HepG2	-	mRNA-seq	hsa-miR-218-5p	100	48
59	GSM3788591	HepG2	-	mRNA-seq	hsa-miR-218-5p	300	48
60	GSM3788592	HepG2	-	mRNA-seq	hsa-miR-218-5p	200	48
61	GSM3788593	HepG2	-	mRNA-seq	hsa-miR-218-5p	200	48
62	-	HepG2	-	mRNA-seq	hsa-miR-218-5p	200	48
63	-	HepG2	-	mRNA-seq	hsa-miR-218-5p	200	48
64	GSM3788594	HepG2	-	mRNA-seq	hsa-miR-216a-5p	100	48
65	GSM3788595	HepG2	-	mRNA-seq	hsa-miR-216a-5p	300	48
66	GSM3788596	HepG2	-	mRNA-seq	hsa-miR-217	100	48
67	GSM3788597	HepG2	-	mRNA-seq	hsa-miR-217	300	48
68	GSM3788598	HepG2	-	mRNA-seq	hsa-miR-217	200	48
69	GSM3788599	HepG2	-	mRNA-seq	hsa-miR-217	200	48
70	GSM3788600	HepG2	-	mRNA-seq	hsa-miR-365a-3p	100	48
71	GSM3788601	HepG2	-	mRNA-seq	hsa-miR-365a-3p	300	48
72	GSM3788605	HepG2	-	mRNA-seq	hsa-miR-1-3p	200	48
73	GSM3788606	HepG2	-	mRNA-seq	hsa-miR-1-3p	300	48
74	GSM3788607	HepG2	-	mRNA-seq	hsa-miR-1-3p	300	48
75	GSM3788611	HepG2	-	mRNA-seq	hsa-miR-142-3p	100	48
76	GSM3788612	HepG2	-	mRNA-seq	hsa-miR-142-3p	300	48
77	GSM3788613	HepG2	-	mRNA-seq	hsa-miR-142-3p	200	48
78	GSM3788614	HepG2	-	mRNA-seq	hsa-miR-142-3p	200	48
79	-	HepG2	-	mRNA-seq	hsa-miR-142-3p	200	48
80	-	HepG2	-	mRNA-seq	hsa-miR-142-3p	200	48
81	GSM3788615	HepG2	-	mRNA-seq	hsa-miR-193b-3p	200	48
82	GSM3788616	HepG2	-	mRNA-seq	hsa-miR-193b-3p	200	48
83	GSM3788617	HepG2	-	mRNA-seq	hsa-miR-142-5p	300	48
84	GSM3788618	HepG2	-	mRNA-seq	hsa-miR-142-5p	200	48
85	GSM3788619	HepG2	-	mRNA-seq	hsa-miR-142-5p	200	48
86	GSM3788620	HepG2	-	mRNA-seq	hsa-miR-451a	300	48
87	GSM3788621	HepG2	-	mRNA-seq	hsa-miR-451a	200	48
88	GSM3788622	HepG2	-	mRNA-seq	hsa-miR-129-2-3p	300	48

89	GSM3788623	HepG2	-	mRNA-seq	hsa-miR-129-2-3p	200	48
90	GSM3788624	HepG2	-	mRNA-seq	hsa-miR-129-2-3p	200	48
91	-	HepG2	-	mRNA-seq	hsa-miR-138-5p	200	48
92	-	HepG2	-	mRNA-seq	hsa-miR-138-5p	200	48
93	-	HepG2	-	mRNA-seq	hsa-miR-138-5p	200	48
94	-	HepG2	-	mRNA-seq	hsa-miR-138-5p	200	48
95	-	HepG2	-	mRNA-seq	hsa-miR-122-5p	200	48
96	-	HepG2	-	mRNA-seq	hsa-miR-122-5p	200	48
97	-	HepG2	-	mRNA-seq	hsa-miR-24-3p	200	48
98	-	HepG2	-	mRNA-seq	hsa-miR-24-3p	200	48
99	-	HepG2	-	mRNA-seq	hsa-miR-126-3p	200	48
100	-	HepG2	-	mRNA-seq	hsa-miR-126-3p	200	48
101	-	HepG2	-	mRNA-seq	hsa-miR-135b-5p	200	48
102	-	HepG2	-	mRNA-seq	hsa-miR-135b-5p	200	48
103	-	HepG2	-	mRNA-seq	hsa-miR-153-3p	200	48
104	-	HepG2	-	mRNA-seq	hsa-miR-153-3p	200	48
105	-	HepG2	-	mRNA-seq	hsa-miR-489-3p	200	48
106	-	HepG2	-	mRNA-seq	hsa-miR-489-3p	200	48
107	-	HepG2	-	mRNA-seq	hsa-miR-145-5p	200	48
108	-	HepG2	-	mRNA-seq	hsa-miR-145-5p	200	48
109	-	HepG2	-	mRNA-seq	hsa-miR-196a-5p	200	48
110	-	HepG2	-	mRNA-seq	hsa-miR-196a-5p	200	48
111	-	HepG2	-	mRNA-seq	hsa-miR-223-3p	200	48
112	-	HepG2	-	mRNA-seq	hsa-miR-223-3p	200	48
113	-	HepG2	-	mRNA-seq	hsa-miR-216b-5p	200	48
114	-	HepG2	-	mRNA-seq	hsa-miR-216b-5p	200	48
115	-	HepG2	-	mRNA-seq	hsa-miR-499a-5p	200	48
116	-	HepG2	-	mRNA-seq	hsa-miR-499a-5p	200	48
117	-	HepG2	-	mRNA-seq	hsa-miR-7-5p	200	48
118	-	HepG2	-	mRNA-seq	hsa-miR-7-5p	200	48
119	-	HepG2	-	mRNA-seq	hsa-miR-187-3p	200	48
120	-	HepG2	-	mRNA-seq	hsa-miR-187-3p	200	48
121	-	HepG2	-	mRNA-seq	hsa-miR-147b	200	48
122	-	HepG2	-	mRNA-seq	hsa-miR-147b	200	48

123	-	HepG2	-	mRNA-seq	hsa-miR-31-5p	200	48
124	-	HepG2	-	mRNA-seq	hsa-miR-31-5p	200	48
125	-	HepG2	-	mRNA-seq	hsa-miR-184	200	48
126	-	HepG2	-	mRNA-seq	hsa-miR-184	200	48
127	GSM3788627	HepG2	-	mRNA-seq	hsa-miR-155-5p	100	48
128	GSM3788628	HepG2	-	mRNA-seq	hsa-miR-155-5p	200	48
129	GSM3788629	HepG2	-	mRNA-seq	hsa-miR-155-5p	300	48
130	GSM3788630	HepG2	-	mRNA-seq	hsa-miR-155-5p	200	48
131	GSM3788631	HepG2	-	mRNA-seq	hsa-miR-155-5p	200	48
132	-	HEK293T	Parental	mRNA-seq	mock	-	24
133	-	HEK293T	Parental	mRNA-seq	mock	-	24
134	-	HEK293T	Parental	mRNA-seq	mock	-	24
135	-	HEK293T	Parental	mRNA-seq	mock	-	24
136	-	HEK293T	Parental	mRNA-seq	mock	-	24
137	-	HEK293T	Parental	mRNA-seq	mock	-	24
138	-	HEK293T	Parental	mRNA-seq	mock	-	24
139	-	HEK293T	PCBP2 KO	mRNA-seq	mock	-	24
140	-	HEK293T	PCBP2 KO	mRNA-seq	mock	-	24
141	-	HEK293T	PCBP2 KO	mRNA-seq	mock	-	24
142	-	HEK293T	PCBP2 KO	mRNA-seq	mock	-	24
143	-	HEK293T	PCBP2 KO	mRNA-seq	mock	-	24
144	-	HEK293T	PCBP2 KO	mRNA-seq	mock	-	24
145	-	HEK293T	IGF2BP1 KO	mRNA-seq	mock	-	24
146	-	HEK293T	IGF2BP1 KO	mRNA-seq	mock	-	24
147	-	HEK293T	IGF2BP1 KO	mRNA-seq	mock	-	24
148	-	HEK293T	IGF2BP1 KO	mRNA-seq	mock	-	24
149	-	HEK293T	IGF2BP1 KO	mRNA-seq	mock	-	24
150	-	HEK293T	IGF2BP1 KO	mRNA-seq	mock	-	24
151	-	HEK293T	IGF2BP1 KO	mRNA-seq	mock	-	24
152	-	HEK293T	Parental	mRNA-seq	hsa-miR-24-3p	100	24
153	-	HEK293T	Parental	mRNA-seq	hsa-miR-31-5p	100	24

154	-	HEK293T	Parental	mRNA-seq	hsa-miR-129-2-3p	75	24
155	-	HEK293T	Parental	mRNA-seq	hsa-miR-199-5p	75	24
156	-	HEK293T	Parental	mRNA-seq	hsa-miR-122-5p	75	24
157	-	HEK293T	Parental	mRNA-seq	hsa-miR-212-5p	75	24
158	-	HEK293T	Parental	mRNA-seq	hsa-miR-455-3p	75	24
159	-	HEK293T	Parental	mRNA-seq	hsa-miR-29a-3p	75	24
160	-	HEK293T	Parental	mRNA-seq	hsa-miR-214-5p	75	24
161	-	HEK293T	Parental	mRNA-seq	hsa-miR-193b-3p	75	24
162	-	HEK293T	PCBP2 KO	mRNA-seq	hsa-miR-24-3p	100	24
163	-	HEK293T	PCBP2 KO	mRNA-seq	hsa-miR-31-5p	100	24
164	-	HEK293T	PCBP2 KO	mRNA-seq	hsa-miR-129-2-3p	75	24
165	-	HEK293T	PCBP2 KO	mRNA-seq	hsa-miR-199-5p	75	24
166	-	HEK293T	PCBP2 KO	mRNA-seq	hsa-miR-122-5p	75	24
167	-	HEK293T	PCBP2 KO	mRNA-seq	hsa-miR-212-5p	75	24
168	-	HEK293T	PCBP2 KO	mRNA-seq	hsa-miR-455-3p	75	24
169	-	HEK293T	PCBP2 KO	mRNA-seq	hsa-miR-214-5p	75	24
170	-	HEK293T	PCBP2 KO	mRNA-seq	hsa-miR-193b-3p	75	24
171	-	HEK293T	IGF2BP1 KO	mRNA-seq	hsa-miR-129-2-3p	75	24
172	-	HEK293T	IGF2BP1 KO	mRNA-seq	hsa-miR-199-5p	75	24
173	-	HEK293T	IGF2BP1 KO	mRNA-seq	hsa-miR-122-5p	75	24
174	-	HEK293T	IGF2BP1 KO	mRNA-seq	hsa-miR-212-5p	75	24
175	-	HEK293T	IGF2BP1 KO	mRNA-seq	hsa-miR-455-3p	75	24
176	-	HEK293T	IGF2BP1 KO	mRNA-seq	hsa-miR-29a-3p	75	24
177	-	HEK293T	IGF2BP1 KO	mRNA-seq	hsa-miR-214-5p	75	24
178	-	HEK293T	IGF2BP1 KO	mRNA-seq	hsa-miR-193b-3p	75	24
179	-	HEK293T	WT	DMS-seq	DMS(-)	-	-
180	-	HEK293T	WT	DMS-seq	DMS(-)	-	-
181	-	HEK293T	WT	DMS-seq	DMS(+)	-	-
182	-	HEK293T	IGF2BP1 KO	DMS-seq	DMS(-)	-	-
183	-	HEK293T	IGF2BP1 KO	DMS-seq	DMS(-)	-	-

184	-	HEK293T	IGF2BP1 KO	DMS-seq	DMS(+)	-	-
185	-	HEK293T	IGF2BP1 KO	DMS-seq	DMS(+)	-	-

Microarrays

No.	Study	Accession	Cell	Treatment	Conc.
1	GSE2075	GSM37599	HeLa	hsa-miR-1-3p	100
2	GSE2075	GSM37601	HeLa	hsa-miR-124-3p	100
3	GSE8501	GSM210897	HeLa	hsa-miR-7-5p	100
4	GSE8501	GSM210898	HeLa	hsa-miR-9-5p	100
5	GSE8501	GSM210901	HeLa	hsa-miR-122-5p	100
6	GSE8501	GSM210903	HeLa	hsa-miR-128-3p	100
7	GSE8501	GSM210904	HeLa	hsa-miR-132-3p	100
8	GSE8501	GSM210907	HeLa	siR-8	100
9	GSE8501	GSM210909	HeLa	hsa-miR-142-3p	100
10	GSE8501	GSM210911	HeLa	hsa-miR-148b-3p	100
11	GSE8501	GSM210913	HeLa	hsa-miR-181a-5p	100
12	Dharmacon2006	16012097017939	HeLa	siR-13	100
13	Dharmacon2006	16012097017952	HeLa	siR-14	100
14	Dharmacon2006	16012097017953	HeLa	siR-15	100
15	Dharmacon2006	16012097016669	HeLa	siR-16	100
16	Dharmacon2006	16012097016668	HeLa	siR-17	100
17	Dharmacon2006	16012097016667	HeLa	siR-18	100
18	Dharmacon2006	16012097016666	HeLa	siR-19	100
19	Dharmacon2006	16012097018568	HeLa	siR-20	100
20	Dharmacon2006	251209725411	HeLa	siR-21	100
21	Dharmacon2008	1595297366	HeLa	siR-22	100
22	Dharmacon2008	1595297383	HeLa	siR-23	100
23	Dharmacon2008	1595297389	HeLa	siR-24	100
24	Dharmacon2008	1595297432	HeLa	siR-29	100
25	Dharmacon2008	1595297491	HeLa	siR-30	100
26	Dharmacon2008	1595297496	HeLa	siR-31	100
27	Dharmacon2008	1595297501	HeLa	siR-32	100
28	Dharmacon2008	1595297507	HeLa	siR-33	100

29	Dharmacon2008	1595297513	HeLa	siR-34	100
30	Dharmacon2008	1595297524	HeLa	siR-36	100
31	Dharmacon2008	1595297530	HeLa	siR-37	100
32	Dharmacon2008	1595297588	HeLa	siR-40	100
33	Dharmacon2008	1595297595	HeLa	siR-41	100
34	Dharmacon2008	1595297605	HeLa	siR-42	100
35	Dharmacon2008	1595297614	HeLa	siR-43	100
36	Dharmacon2008	1595297621	HeLa	siR-44	100
37	Dharmacon2008	1595297627	HeLa	siR-45	100
38	Dharmacon2008	1595297644	HeLa	siR-46	100
39	Dharmacon2008	1595297650	HeLa	siR-47	100
40	GSE5291	GSM119708	HeLa	siR-50	100
41	GSE5291	GSM119710	HeLa	siR-51	100
42	GSE5291	GSM119743	HeLa	siR-52	100
43	GSE5291	GSM119749	HeLa	siR-56	100
44	GSE5291	GSM119759	HeLa	siR-58	100
45	GSE5291	GSM119762	HeLa	siR-60	100
46	GSE5291	GSM119763	HeLa	siR-61	100
47	GSE5769	GSM134325	HeLa	siR-62	100
48	GSE5769	GSM134327	HeLa	siR-63	100
49	GSE5769	GSM134551	HeLa	siR-64	100
50	GSE5814	GSM133685	HeLa	siR-65	100
51	GSE5814	GSM133689	HeLa	siR-66	100
52	GSE5814	GSM133699	HeLa	siR-67	100
53	GSE5814	GSM133700	HeLa	siR-68	100
54	GSE5814	GSM134466	HeLa	siR-69	100
55	GSE5814	GSM134480	HeLa	siR-70	100
56	GSE5814	GSM134483	HeLa	siR-71	100
57	GSE5814	GSM134485	HeLa	siR-72	100
58	GSE5814	GSM134511	HeLa	siR-73	100
59	GSE5814	GSM134512	HeLa	siR-74	100
60	GSE56243	GSM1357597	PC3	hsa-miR-218	10
61	GSE56243	GSM1357599	FaDu	hsa-miR-144	10
62	GSE56243	GSM1357603	DU145	hsa-miR-221	10

63	GSE56243	GSM1357604	DU145	hsa-miR-222	10
64	GSE56243	GSM1357608	PC3	hsa-miR-224	10
65	GSE56243	GSM1357615	PC3	hsa-miR-452	10
66	GSE66498	GSM1623420	PC3	hsa-miR-205	10
67	GSE66498	GSM1623421	DU145	hsa-miR-205	10
68	GSE66498	GSM1623423	786-O	hsa-miR-29a	10
69	GSE66498	GSM1623424	A498	hsa-miR-29a	10
70	GSE66498	GSM1623425	T24	hsa-miR-451	10
71	GSE66498	GSM1623426	T24	hsa-miR-144-3p	10
72	GSE66498	GSM1623427	T24	hsa-miR-144-5p	10
73	GSE66498	GSM1623436	BOY	hsa-miR-145-5p	10
74	GSE66498	GSM1623437	BOY	hsa-miR-145-3p	10
75	GSE66498	GSM1623438	T24	hsa-miR-145-5p	10
76	GSE66498	GSM1623439	T24	hsa-miR-145-3p	10
77	GSE66498	GSM1623444	PC3	hsa-miR-221	10
78	GSE66498	GSM1623445	PC3	hsa-miR-222	10
79	GSE66498	GSM1623446	PC3	hsa-miR-223	10
80	GSE77790	GSM2059405	EBC-1	hsa-miR-145-3p	10
81	GSE77790	GSM2059406	EBC-1	hsa-miR-218	10
82	GSE77790	GSM2059409	PC3	hsa-miR-320a	10
83	GSE77790	GSM2059419	BOY	hsa-miR-218	10
84	GSE77790	GSM2059427	PC3	hsa-miR-29a	10
85	GSE77790	GSM2059434	PC3	hsa-miR-145-5p	10
86	GSE82108	GSM2183551	FaDu	hsa-miR-150-5p	10
87	GSE82108	GSM2183552	FaDu	hsa-miR-150-3p	10
88	GSE82108	GSM2183555	EBC-1	hsa-miR-150-5p	10
89	GSE82108	GSM2183556	EBC-1	hsa-miR-150-3p	10
90	GSE82108	GSM2183557	PANC1	hsa-miR-216b-3p	10
91	GSE82108	GSM2183558	SW1990	hsa-miR-216b-3p	10
92	GSE85614	GSM2279807	PC3	hsa-miR-150-5p	10
93	GSE85614	GSM2279808	PC3	hsa-miR-150-3p	10
94	GSE85614	GSM2279809	PC3	hsa-miR-99a-3p	10
95	GSE85614	GSM2279810	PC3	hsa-miR-100-3p	10
96	GSE93290	GSM2450420	A498	hsa-miR-10a-5p	10

97	GSE93290	GSM2450421	786-O	hsa-miR-10a-5p	10
98	GSE93290	GSM2450422	A549	hsa-miR-150-5p	10
99	GSE93290	GSM2450423	A549	hsa-miR-150-3p	10
100	GSE93290	GSM2450424	TE8	hsa-miR-150-5p	10
101	GSE93290	GSM2450425	TE8	hsa-miR-150-3p	10
102	GSE93290	GSM2450428	PANC1	hsa-miR-148a-5p	10
103	GSE93290	GSM2450429	PANC1	hsa-miR-148a-3p	10
104	GSE93290	GSM2450430	SW1990	hsa-miR-148a-5p	10
105	GSE93290	GSM2450431	SW1990	hsa-miR-148a-3p	10
106	GSE93290	GSM2450432	PC3	hsa-miR-499a-5p	10
107	GSE93290	GSM2450434	PC3	hsa-miR-455-3p	10
108	GSE100746	GSM2692407	A498	hsa-miR-149-5p	10
109	GSE100746	GSM2692408	A498	hsa-miR-149-3p	10
110	GSE100746	GSM2692409	PC3	hsa-miR-455-5p	10
111	GSE100746	GSM2692410	PC3	hsa-miR-455-3p	10
112	GSE106791	GSM2850316	786-O	hsa-miR-455-3p	10
113	GSE106791	GSM2850317	786-O	hsa-miR-455-5p	10
114	GSE106791	GSM2850322	PC3	hsa-miR-184	10
115	GSE106791	GSM2850323	PC3	hsa-miR-30a-5p	10
116	GSE106791	GSM2850324	PC3	hsa-miR-30a-3p	10
117	GSE106791	GSM2850325	T24	hsa-miR-107	10
118	GSE106791	GSM2850326	T24	hsa-miR-185-5p	10
119	GSE106791	GSM2850327	T24	hsa-miR-320b	10
120	GSE106791	GSM2850328	T24	hsa-miR-320c	10
121	GSE106791	GSM2850329	PC3	hsa-miR-199-3p	10
122	GSE107008	GSM2859381	T24	hsa-miR-223-3p	10
123	GSE107008	GSM2859382	T24	hsa-miR-223-5p	10
124	GSE107008	GSM2859385	A549	hsa-miR-145-5p	10
125	GSE107008	GSM2859387	TE8	hsa-miR-145-5p	10
126	GSE107008	GSM2859388	TE8	hsa-miR-145-3p	10
127	GSE115800	GSM3190195	PC3	hsa-miR-125b-1-3p	10
128	GSE115800	GSM3190196	786-O	hsa-miR-532-5p	10
129	GSE115800	GSM3190198	T24	hsa-miR-140-5p	10
130	GSE115800	GSM3190199	T24	hsa-miR-140-3p	10

131	GSE115801	GSM3190204	SW1990	hsa-miR-204-5p	10
132	GSE118966	GSM3355022	786-O	hsa-miR-101-3p	10
133	GSE118966	GSM3355023	786-O	hsa-miR-184	10
134	GSE118966	GSM3355024	A498	hsa-miR-184	10

Supplementary Table 3. Synthetic miRNA duplexes for miRNA overexpression experiments

Synthetic miRNA duplexes were designed for the overexpression of the corresponding miRNA. Mature miRNA symbol name, seed with m8 sequence and guide/passenger sequences (5′ to 3′) were listed.

No.	mature name	seed+m8	guide strand	passenger strand
1	hsa-miR-211-5p	UCCCUUU	UUCCCUUUGUCAUCCUUCGCCU	GCGAAGGAUGACAAAGGCCAUA
2	hsa-miR-18a-5p	AAGGUGC	UAAGGUGCAUCUAGUGCAGAUAG	AUCUGCACUAGAUGCACCUCAUA
3	hsa-miR-137	UAUUGCU	UUAUUGCUUAAGAAUACGCGUAG	ACGCGUAUUCUUAAGCAAUCAUA
4	hsa-miR-140-5p	AGUGGUU	CAGUGGUUUUACCCUAUGGUAG	ACCAUAGGGUAAAACCAGAGUA
5	hsa-miR-133a-3p	UUGGUCC	UUUGGUCCCCUUCAACCAGCUG	GCUGGUUGAAGGGGACCACAUA
6	hsa-miR-101-3p	ACAGUAC	UACAGUACUGUGAUAACUGAA	CAGUUAUCACAGUACUACAUA
7	hsa-miR-140-3p	ACCACAG	UACCACAGGGUAGAACCACGG	GUGGUUCUACCCUGUGACAUA
8	hsa-miR-199b-3p	CAGUAGU	ACAGUAGUCUGCACAUUGGUUA	ACCAAUGUGCAGACUACUAUUA
9	hsa-miR-218-5p	UGUGCUU	UUGUGCUUGAUCUAACCAUGU	AUGGUUAGAUCAAGCAGGAUA
10	hsa-miR-216a-5p	AAUCUCA	UAAUCUCAGCUGGCAACUGUGA	ACAGUUGCCAGCUGAGAUCAUA
11	hsa-miR-217	ACUGCAU	UACUGCAUCAGGAACUGAUUGGA	CAAUCAGUUCCUGAUGCACCAUA
12	hsa-miR-365a-3p	AAUGCCC	UAAUGCCCCUAAAAAUCCUUAU	AAGGAUUUUUAGGGGCAUCAUA
13	hsa-miR-1-3p	GGAAUGU	UGGAAUGUAAAGAAGUAUGUAU	ACAUACUUCUUUACAUUAGAUA
14	hsa-miR-142-3p	GUAGUGU	UGUAGUGUUUCCUACUUUAUGGA	CAUAAAGUAGGAAACACUAGAUA
15	hsa-miR-193b-3p	ACUGGCC	AACUGGCCCUCAAAGUCCCGCU	CGGGACUUUGAGGGCCACAUUA
16	hsa-miR-142-5p	AUAAAGU	CAUAAAGUAGAAAGCACUACU	UAGUGCUUUCUACUUUACGUA
17	hsa-miR-451a	AACCGUU	AAACCGUUACCAUUACUGAGUU	CUCAGUAAUGGUAACGGUAUUA
18	hsa-miR-129-2-3p	AGCCCUU	AAGCCCUUACCCCAAAAAGCAU	GCUUUUUGGGGUAAGGGAAUUA
19	hsa-miR-138-5p	GCUGGUG	AGCUGGUGUUGUGAAUCAGGCCG	GCCUGAUUCACAACACCAUAUUA
20	hsa-miR-122-5p	GGAGUGU	UGGAGUGUGACAAUGGUGUUUG	AACACCAUUGUCACACUAGAUA
21	hsa-miR-24-3p	GGCUCAG	UGGCUCAGUUCAGCAGGAACAG	GUUCCUGCUGAACUGAGAGAUA
22	hsa-miR-126-3p	CGUACCG	UCGUACCGUGAGUAAUAAUGCG	CAUUAUUACUCACGGUAUUAUA
23	hsa-miR-135b-5p	AUGGCUU	UAUGGCUUUUCAUUCCUAUGUGA	ACAUAGGAAUGAAAAGCCACAUA
24	hsa-miR-153-3p	UGCAUAG	UUGCAUAGUCACAAAAGUGAUC	UCACUUUUGUGACUAUGUGAUA
25	hsa-miR-489-3p	UGACAUC	GUGACAUCACAUAUACGGCAGC	UGCCGUAUAUGUGAUGUGGCUA
26	hsa-miR-145-5p	UCCAGUU	GUCCAGUUUUCCCAGGAAUCCCU	GGAUUCCUGGGAAAACUGCUCUA
27	hsa-miR-196a-5p	AGGUAGU	UAGGUAGUUUCAUGUUGUUGGG	CAACAACAUGAAACUACAGAUA
28	hsa-miR-223-3p	GUCAGUU	UGUCAGUUUGUCAAAUACCCCA	GGGUAUUUGACAAACUGAGAUA

29	hsa-miR-216b-5p	AAUCUCU	AAAUCUCUGCAGGCAAAUGUGA	ACAUUUGCCUGCAGAGAUAUUA
30	hsa-miR-499a-5p	UAAGACU	UUAAGACUUGCAGUGAUGUUU	ACAUCACUGCAAGUCUUCAUA
31	hsa-miR-7-5p	GGAAGAC	UGGAAGACUAGUGAUUUUGUUGU	AACAAAAUCACUAGUCUUAGAUA
32	hsa-miR-187-3p	CGUGUCU	UCGUGUCUUGUGUUGCAGCCGG	GGCUGCAACACAAGACAUUAUA
33	hsa-miR-147b	UGUGCGG	GUGUGCGGAAAUGCUUCUGCUA	GCAGAAGCAUUUCCGCAGGCUA
34	hsa-miR-31-5p	GGCAAGA	AGGCAAGAUGCUGGCAUAGCU	CUAUGCCAGCAUCUUGAAUUA
35	hsa-miR-184	GGACGGA	UGGACGGAGAACUGAUAAGGGU	CCUUAUCAGUUCUCCGUAGAUA
36	hsa-miR-155-5p	UAAUGCU	UUAAUGCUAAUCGUGAUAGGGGU	CCCUAUCACGAUUAGCAUUCAUA

Supplementary Table 4. List of primers used in the study

The primers used for generation of recombinant proteins, qPCR, knockout genotyping, rescue experiment, EMSA and luciferase assays. The mutated regions are highlighted in red.

Cloning of pET-28b plasmid for recombinant protein

Name	Sequence (5′ to 3′)
FW: FUBP3 CDS	CCGCGCGGCAGCCATATGGCGGAGCTGGTGCAGGGGCA
RV: FUBP3 CDS	GTGGTGGTGCTCGAGCTACTGCTCCTGGCTGTGGGCCT
FW: PCBP2 CDS	CCGCGCGGCAGCCATATGGACACCGGTGTGATTGAA
RV: PCBP2 CDS	GTGGTGGTGCTCGAGCTAGCTGCTCCCCATGCCA

Quantitative PCR

Name	Sequence (5′ to 3′)
FW: KATNA1	GTCCATGCCTGTACCTGTTGA
RV: KATNA1	CGAACAGCTTTCCCTCTGTCA
FW: UBA1	GTGATCCCCTTCCTGACAGA
RV: UBA1	GACATTTTCTGCTGGCTGCTT
FW: FARSA	CGACACCTTCTTCCTTCGAGA
RV: FARSA	GTGTGGGTTCGCAGTAGGTT
FW: ACSF2	GAACCCAGCCTACCAGGCTAT
RV: ACSF2	CAGATCTGGGAGCCTCTGACT
FW: DYNLRB1	CAGGGAATCATCGTCGTGAACA
RV: DYNLRB1	CGAATTCGAAGGAAGGTGAGA
FW: HGS	GGTCCAGGACACCTACCAGA
RV: HGS	AGTGGTGCTTACGGGTCATC
FW: EPHX1	CTCAGAGGCATCCTCCAAGAA
RV: EPHX1	GAGGAGGGTCAGGGTAGAGAA

Knockout genotyping

Name	Sequence (5′ to 3′)
FW: PCBP2 KO	GTCATGCAGTTGTGCTTGGTGT
RV: PCBP2 KO	GCTCCAAGATGCATGTCTGCTCT
FW: IGF2BP1 KO	AAAGAAAGTTTGCGGCTCCT
RV: IGF2BP1 KO	GTAGCCGGATTTGACCAAGA

Cloning of pcDNA3.1 vector for rescue experiment

Name	Sequence (5′ to 3′)
FW2: PCBP2 CDS	AAGCTTATGGACACCGGTGTGATTGAA
RV2: PCBP2 CDS	CTCGAGCTAGCTGCTCCCCATGCCA
FW: IGF2BP1 CDS	GACGATGACGATAAAAACAAGCTTTACATCGGCAACCTCA
RV: IGF2BP1 CDS	GTGGCGGCCGCTCGATCACTTCCTCCGTGCCTGGGCCT

EMSA

Name	Sequence (5′ to 3′)
FW: T7_SSB	TAATACGACTCACTATAGGGGAGAAAGGAAAAATTTTTTTGTTGT
RV: SSB	TGATATTTGAATCATCTGAAATAACA
FW: T7_UBA1	TAATACGACTCACTATAGGGCCCAACTAGCCAAGTCTGGT
RV: UBA	TGGGAGTTATTAATTCTTTATTAGGA
FW: T7_CRAT_WT	TAATACGACTCACTATAGGGCCTTGGTTCCCTCTTCCTT
FW: T7_CRAT_MUT	TAATACGACTCACTATAGGGCCTTGGTTCCCTCTTCAGAA
RV: CRAT	ACGGAAGGCACTTGGCT

DMS-seq

Name	Sequence (5′ to 3′)
3′ adaptor	5′-/rApp/NNNNTGGAATTCTCGGGTGCCAAGG/3ddC/-3′
5′ adaptor	5′-[Phosphate]NNNNGATCGTCGGACTGTAGAACTCTGAAC[C3 spacer]-3′

Luciferase reporter assay (HepG2)

Name	Sequence (5′ to 3′)
FW: SSB_RBS^{WT}MTS^{WT}	TAGGCGATCGCTCGATTTAGTAAACCAATTTTTTATTCATTTT
RV: SSB_RBS^{WT}MTS^{WT}	AATTCCCGGGCTCGATTGCTCTTTTCATATAGTATATATTTTA
FW: SSB_RBS^{WT}MTS^{MUT}	CAAA**TATGAGT**TCTTTGAATGTATTGTTCTGTTTGT
RV: SSB_RBS^{WT}MTS^{MUT}	AAGA**ACTCATA**TTTGCATAACAAAAAGACAAGTTAA
FW: SSB_RBS^{MUT}MTS^{WT}	**TGA**CAAATGAGATTTC**TATTG**ATGTATTGTTCTGTTTGTGTTA
RV: SSB_RBS^{MUT}MTS^{WT}	**CAATA**GAAATCTCATTTG**TCA**AACAAAAAGACAAGTTAAACAACA
FW: SSB_RBS^{MUT}MTS^{MUT}	CAAA**TATGAGT**TC**TATTG**ATGTATTGTTCTGTTTGT
RV: SSB_RBS^{MUT}MTS^{MUT}	**TA**GA**ACTCATA**TTTG**TCAAA**CAAAAAGACAAGTTAA

FW: LMAN2_RBS^{WT}MTS^{WT}<br>RV: LMAN2_RBS^{WT}MTS^{WT}	TAGGCGATCGCTCGACCCAGGAGCCAATGTGAACT<br>AATTCCCGGGCTCGATGAGGTTGGGTATTTCACTTTAT
FW: LMAN2_RBS^{WT}MTS^{MUT}<br>RV: LMAN2_RBS^{WT}MTS^{MUT}	TTTTGCTTTTGCCCCAGCAGGGACAGGTGGCA<br>GGGGCAAAAGCAAAATGTATGAAAATACTTTAAT
FW: LMAN2_RBS^{MUT}MTS^{WT}<br>RV: LMAN2_RBS^{MUT}MTS^{WT}	CTTTGTCTTGCCCAGCGGAAGCAGGTGGCAGAGCCGAGGCT<br>CTTCCGCTGGGCAAGACAAAGAATGTATGAAAATACTTTAATCATTTA
FW: LMAN2_RBS^{MUT}MTS^{MUT}<br>RV: LMAN2_RBS^{MUT}MTS^{MUT}	TTCTTTGTTTGCCCCAGCGGAAGCAGGTGGCA<br>GGGGCAAACAAAGAATGTATGAAAATACTTTAAT
FW: UBA1_RBS^{WT}MTS^{WT} (miR-31-5p)<br>RV: UBA1_RBS^{WT}MTS^{WT} (miR-31-5p)	TAGGCGATCGCTCGACCCCGTCTGCTCCTCTAG<br>AATTCCCGGGCTCGATGGGAGTTATTAATTCTTTATTAGGA
FW: UBA1_RBS^{WT}MTS^{MUT} (miR-31-5p)<br>RV: UBA1_RBS^{WT}MTS^{MUT} (miR-31-5p)	CCCCTTTGCCCACTGCCTTCTACCTTGTT<br>CAGTGGGCAAAGGGGTTCAGGTAGGGGGA
FW: UBA1_RBS^{WT}MTS^{MUT} (miR-34a-5p)<br>RV: UBA1_RBS^{WT}MTS^{MUT} (miR-34a-5p)	CTTGCCCCTAGCTTCTACCTTGTTTGAAACCTGA<br>GAAGCTAGGGGCAAGAGGGGTTCAGGTA
FW: UBA1_RBS^{MUT}MTS^{WT} (miR-31-5p)<br>RV: UBA1_RBS^{MUT}MTS^{WT} (miR-31-5p)	ATCCTATCCCCCTACGACATCCCCTCTTGCCACTGCCTTCTA<br>ATGTCGTAGGGGGATAGGATGGGAACACCAGACTTGGCTAGT
FW: UBA1_RBS^{MUT}MTS^{MUT} (miR-31-5p)<br>RV: UBA1_RBS^{MUT}MTS^{MUT} (miR-31-5p)	CCCCTTTGCCCACTGCCTTCTACCTTGTT<br>CAGTGGGCAAAGGGGATGTCGTAGGGGGA
FW: UBA1_RBS^{MUT}MTS^{MUT} (miR-34a-5p)<br>RV: UBA1_RBS^{MUT}MTS^{MUT} (miR-34a-5p)	CTTGCCCCTAGCTTCTACCTTGTTTGAAACCTGA<br>GAAGCTAGGGGCAAGAGGGGATGTCGTA
FW: MGAT4B_RBS^{WT}MTS^{WT}<br>RV: MGAT4B_RBS^{WT}MTS^{WT}	TAGGCGATCGCTCGAGCTTCTGAGGGTACCCTGT<br>AATTCCCGGGCTCGAGAGGCACACACTTCATTAACCCT
FW: MGAT4B_RBS^{WT}MTS^{MUT}<br>RV: MGAT4B_RBS^{WT}MTS^{MUT}	GCGAACAGCTGAATGCATATACTACTTTATGTGCTGTGT<br>CATTCAGCTGTTCGCGCCAGAGACGG
FW: MGAT4B_RBS^{MUT}MTS^{WT}<br>RV: MGAT4B_RBS^{MUT}MTS^{WT}	AGCTATTCTGGTGTCGCCCGCCGTCTCTGGCGCGA<br>GGCGACACCAGAATAGCTCTTCTAAAACGGCCTGACT
FW: MGAT4B_RBS^{MUT}MTS^{MUT}<br>RV: MGAT4B_RBS^{MUT}MTS^{MUT}	GCGAACAGCTGAATGCATATACTACTTTATGTGCTGTGT<br>CATTCAGCTGTTCGCGCCAGAGACGG
FW: CMSS1_RBS^{WT}MTS^{WT}<br>RV: CMSS1_RBS^{WT}MTS^{WT}	TAGGCGATCGCTCGAGTCTGTGTCCTAATGAAGATTCCA<br>AATTCCCGGGCTCGAACAGAAAAACACGGTCCGCT
FW: CMSS1_RBS^{WT}MTS^{MUT}<br>RV: CMSS1_RBS^{WT}MTS^{MUT}	TTGCAACCATGCAGTAAATATCAGCAAATAGTTTAT<br>ACTGCATGGTTGCAATGTATCAGAGTCATTAAAT
FW: CMSS1_RBS^{MUT}MTS^{WT}<br>RV: CMSS1_RBS^{MUT}MTS^{WT}	TATACAGCAAATAGTTTATGTTATAGTTGTCAACAGATGGATCACT<br>ACTATAACATAAACTATTTGCTGTATATTACTGAGTGCTTGCAATGT

FW: CMSS1_RBS^{MUT}MTS^{MUT}<br>RV: CMSS1_RBS^{MUT}MTS^{MUT}	TTGC**AACCATG**CAGTAA**TATAC**AGCAAATAGTTTAT<br>ACTG**CATGGTT**GCAATGTATCAGAGTCATTAAAT
FW: PLOD3_RBS^{WT}MTS^{WT}<br>RV: PLOD3_RBS^{WT}MTS^{WT}	TAGGCGATCGCTCGACACTCAACCACTCTGCCAAA<br>AATTCCCGGGCTCGACAAGACTTACAAACTGTCCTTT
FW: PLOD3_RBS^{WT}MTS^{WT}<br>RV: PLOD3_RBS^{WT}MTS^{WT}	CATT**CTGCTGT**TTTAGGGGGCCTGGCCC<br>TAAA**ACAGCAG**AATGGCAGGGCAGGTTTG
FW: PLOD3_RBS^{WT}MTS^{WT}<br>RV: PLOD3_RBS^{WT}MTS^{WT}	GC**CCTGG**CC**CTCCG**CTGGGAGTTGGGGGATGGGT<br>G**CGGAG**GG**CCAGG**GCCCCCTAAAAAGGCACA
FW: PLOD3_RBS^{WT}MTS^{WT}<br>RV: PLOD3_RBS^{WT}MTS^{WT}	CATT**CTGCTGT**TTTAGGGGGC**CCTGG**CC<br>TAAA**ACAGCAG**AATGGCAGGGCAGGTTTG
FW: SEC61G_ RBS^{WT}MTS^{WT}<br>RV: SEC61G_ RBS^{WT}MTS^{WT}	TAGGCGATCGCTCGAATACATTTTGGAAGAGAGTTTTTCA<br>AATTCCCGGGCTCGAATTCATTTACTTTGAAATTACTTTAATT
FW: SEC61G_ RBS^{WT}MTS^{MUT}<br>RV: SEC61G_ RBS^{WT}MTS^{MUT}	CATT**CTGCTGT**TTTAGGGGGCCTGGCCC<br>TAAA**ACAGCAG**AATGGCAGGGCAGGTTTG
FW: SEC61G_ RBS^{MUT}MTS^{WT}<br>RV: SEC61G_ RBS^{MUT}MTS^{WT}	**ATTTC**CTTAGAGATTG**GAATG**CAAGTGTGAGGGTGTGAGA<br>**CATTC**CAATCTCTAAG**GAAAT**AAACTCTCTTCCAAAATGTAT
FW: SEC61G_ RBS^{MUT}MTS^{MUT}<br>RV:SEC61G_ RBS^{MUT}MTS^{MUT}	CATT**CTGCTGT**TTTAGGGGGC**CCTGG**CC<br>TAAA**ACAGCAG**AATGGCAGGGCAGGTTTG
FW: CFDP1_ RBS^{WT}MTS^{WT}<br>RV: CFDP1_ RBS^{WT}MTS^{WT}	TAGGCGATCGCTCGATGTTACGGGCTAAATCAAGA<br>AATTCCCGGGCTCGACATCTTCATTATCCTCTCACA
FW: CFDP1_ RBS^{WT}MTS^{MUT}<br>RV: CFDP1_ RBS^{WT}MTS^{MUT}	TTTA**CTGTAGT**TCTCATCATCTGTTTCCCA<br>GAGA**ACTACAG**TAAAACATTTCACAGACGCA
FW: CFDP1_ RBS1^{MUT}MTS^{WT}<br>RV: CFDP1_ RBS1^{MUT}MTS^{WT}	TTTCT**CACAT**TCTGTTTCCCAGCAAGGTCT<br>ACAGA**ATGTG**AGAAACACTGTAAAACATTTCA
FW: CFDP1_ RBS2^{MUT}MTS^{WT}<br>RV: CFDP1_ RBS2^{MUT}MTS^{WT}	TTTTT**ACTTT**CATTGAAGTTCTGTCTATGTATCT<br>CAATG**AAAGT**AAAAAAAAGACCTTGCTGGGAA
FW: CFDP1_ RBS^{MUT}MTS^{MUT}<br>RV: CFDP1_ RBS^{MUT}MTS^{MUT}	TTTA**CTGTAGT**TCT**CACAT**TCTGTTTCCCA<br>GAGA**ACTACAG**TAAAACATTTCACAGACGCA
FW: PHGDH_ RBS^{WT}MTS^{WT}<br>RV: PHGDH_ RBS^{WT}MTS^{WT}	TAGGCGATCGCTCGACCTTGGAGCTCACTGGTCCCT<br>AATTCCCGGGCTCGAGCAGAAGGAGTTGGGGGTAGGCT
FW: PHGDH_ RBS^{WT}MTS^{MUT}<br>RV: PHGDH_ RBS^{WT}MTS^{MUT}	GTAC**AACATGA**ACCGTCTAATAAAGAGCCT<br>CGGT**TCATGTT**GTACTACAGGGTCAGAGT
FW: PHGDH_ RBS^{MUT}MTS^{WT}<br>RV: PHGDH_ RBS^{MUT}MTS^{WT}	CCT**ACCTG**A**CCTTG**TACACTGCACTCTGACCCTGT<br>A**CAAGG**T**CAGGT**AGGCCCGCGTTCAGCCCAA

FW: PHGDH_ RBS^{MUT}MTS^{MUT}<br>RV: PHGDH_ RBS^{MUT}MTS^{MUT}	GTAC**AACATGA**ACCGTCTAATAAAGAGCCT<br>CGGT**TCATGTT**GTACTACAGGGTCAGAGT

Luciferase reporter assay (HEK293T, RBP knockout)

**Name**	**Sequence (5′ to 3′)**
FW: FARSA_MTS^{WT}<br>RV: FARSA_MTS^{WT}	TAGGCGATCGCTCGACATGGGCCACTCTAGGACAGGT<br>AATTCCCGGGCTCGAGCCACCAGATGGGGGCAACT
FW: FARSA_MTS^{MUT}<br>RV: FARSA_MTS^{MUT}	CCCT**AGCCTGG**GACCTTGTATTTATGAGGCCT<br>GGTC**CCAGGCT**AGGGATGCAAAGGAGCGCA
FW: ACSF2_MTS^{WT}<br>RV: ACSF2_MTS^{WT}	TAGGCGATCGCTCGAATAAAGCAGCAGGCCTGTCCT<br>AATTCCCGGGCTCGAAGCAAGAAGCCAAAGAAAACAGA
FW: ACSF2_MTS^{MUT}<br>RV: ACSF2_MTS^{MUT}	CAAG**AGGCCTA**AAGGCAGGCAGCCTGCCCA<br>CCTT**TAGGCCT**CTTGTGCCCAGGCGAGTT
FW: HGS_MTS^{WT}<br>RV: HGS_MTS^{WT}	TAGGCGATCGCTCGACCCAGGCCATGCTCACGT<br>AATTCCCGGGCTCGACGCTGTACCATTCTGGGGCA
FW: HGS_MTS^{MUT}<br>RV: HGS_MTS^{MUT}	AGTA**ACACCTA**ATACAGTTCACCTGAAACGCCT<br>GTAT**TAGGTGT**TACTCCGGACGTGAGCAT
FW: DYNLRB1_MTS^{WT}<br>RV: DYNLRB1_MTS^{WT}	TAGGCGATCGCTCGAGCCACTCTCTTGGCTCCCT<br>AATTCCCGGGCTCGAAAACAGCTTTCTCCAAGGGCGA
FW: DYNLRB1_MTS^{MUT}<br>RV: DYNLRB1_MTS^{MUT}	ACTT**CCTGATG**TTGGAGCAAGAGCTTGCA<br>CCAA**CATCAGG**AAGTGAGGAGAGGGCGCT
FW: EPHX1_MTS^{WT}<br>RV: EPHX1_MTS^{WT}	TAGGCGATCGCTCGACACAAGTGCCCTCCAGGCTT<br>AATTCCCGGGCTCGACATCAAAGCCATGTTGCTTACCA
FW: EPHX1_MTS^{MUT}<br>RV: EPHX1_MTS^{MUT}	TGCC**TCCTCCG**CCTGCCCATGCTGGGA<br>CAGG**CGGAGGA**GGCAAACTCATTCCTCAGAAA

Supplementary Methods

Detection of crosslinking-induced truncation sites

To identify more precise positions of the RBP-binding sites (RBSs) and quantify the RBP-binding signals, we incorporated crosslinking-induced truncation sites (CITSs)[7]. CITS is the mRNA position where reverse transcription is early terminated due to UV crosslinking between an RNA and an RBP in CLIP-seq data and can be detected by mapping the 5′ end of a sequenced reverse read. From the ENCODE database, we obtained eCLIP-seq raw data and the corresponding size-matched normal data aligned to human genome (hg38) for each RBP. Positions of the 5′ ends of the reverse reads were collected and the number of reads mapped on each 5′ end position was counted. When the 3′ end of a forward read is located within 10 nts from the 5′ end of the paired reverse read, the 3′ end position was also counted. The counted numbers of the CITS reads were normalized by 10 million mapped CITS reads and the average number was obtained where multiple replicates are available. Similarly, the normalized number of the CITS reads were obtained for the corresponding size-matched normal data. By subtracting the size-matched normal count from the normalized CITS count, we obtained the normal-controlled CITS count for each mRNA position and utilized for further analyses.

Quantification of cytoplasmic fraction of RBPs

The quantification of cytoplasmic fraction for each RBP was performed for HepG2 and HeLa cell lines using the images from immuno-fluorescence experiments performed by the RBP Image Database[8] (http://rnabiology.ircm.qc.ca/RBPImage/). For HepG2 cell line, 6,315 images were used to quantitatively assess the localization of 104 available RBPs and for HeLa cell line, 5,975 images were used to assess localizations of 102 available RBPs. Each image is a result from co-labeling of three components: RBP (green), DAPI (blue), and specific subcellular organelle marker (red). From each image, using the ImageJ software[9], three channels were separated and the background was subtracted from the green channel. Then, the nuclei were localized by the blue channel. After selecting the nucleus and subtracting the background from the green channel, the intensity was measured for cytoplasm and nucleus regions.

The cytoplasmic fraction of the fluorescence intensity of each RBP compared to that of the nucleus was calculated using the following formula:

$$\text{Fraction of } intensity_{cyt} = \frac{intensity_{cyt}}{intensity_{nuc} + intensity_{cyt}} \qquad (1)$$

The cytoplasmic fractions of fluorescence intensity values were averaged from all available images for each RBP (**Supplementary Fig. 1b**).

Library preparation

For transcriptome library preparation, HepG2 cells and HEK293T cells (Parental, *PCBP2* KO, and *IGF2BP1* KO) were seeded on a 60 mm plate and 100 mm plate respectively. Both cells were transfected at 24 hours after seeding using designed miRNA (100-300 nM) and Lipofectamine RNAiMax (Invitrogen). After 48 and 24 hours of incubation, cells were lysed by using lysis buffer (adapted from the Bartel lab's lysis protocol[10]). For transcriptome library preparation of the HCT116 cell line, cells were lysed using the same protocol mentioned above except that transfection was not performed. Briefly, pelleted cells were lysed by treating with lysis buffer (10 mM Tris-HCl (pH 7.4), 5 mM $MgCl_2$, 100 mM KCl, 1% Triton X-100) and incubating for 10 min at 4°C. From the lysed supernatant, total RNAs were extracted using Trizol according to the manufacturer's protocol (Qiagen). After total RNA extraction, mRNAs were isolated using Dynabead following manufacturer's protocol (Invitrogen). Isolated mRNAs were randomly fragmented using the Fragmentation buffer (Invitrogen). After fragmenting for 2.5 min, to remove residual buffer, the fragmented RNAs were treated with RNeasy MinElute clean up kit (Qiagen).

For sRNA-seq library preparation (HeLa, HepG2, HCT116, and HEK293T cell lines), total miRNAs were extracted from cells using Pure link miRNA isolation Kit (Invitrogen). For both transcriptome and sRNA-seq library construction, TruSeq Small RNA Library Prep Kit (Illumina) was used with following modifications. After removing phosphate groups at 3′ end using Antarctic Phosphatase (NEB) at 37°C for 60 min, the fragmented mRNAs were phosphorylated using T4 polynucleotide kinase (NEB) at 37°C for 60 min. Then, mRNAs were size-selected on a 5% UREA polyacrylamide gel. The size-selected products were then ligated with 3′ and 5′ adapters using T4 RNA ligase 2 truncated (NEB) and T4 RNA ligase 1 (NEB) respectively. After ligating 3′ adapter for 60 min at 28°C, the ligation was stopped using stop solution

(Illumina) and then 5′ adapter was subsequently ligated for 60 min at 28°C. The adapter ligated samples were reverse transcribed using Superscript III (Invitrogen) according to the manufacturer's protocol (50°C, 60 min), and PCR amplified using PML, RP1, and RPIX from TruSeq Small RNA Library Prep Kit (Illumina). The amplified cDNA products were then gel-purified using 5% polyacrylamide gel (BIORAD). The HepG2, HEK293T (Parental, *PCBP2* KO, and *IGF2BP1* KO), and HCT116 mRNA transcriptome libraries were sequenced using Hiseq 2500 and the HeLa, HepG2, HCT116 and HEK293T sRNA-seq libraries were sequenced using Hiseq 2000 (single-end, 51 bp, Illumina).

Selection of reference mRNAs

We obtained the UCSC refFlat annotation (hg38) curated from NCBI's RefSeq database for human genes[11] to collect a reference mRNA set. To obtain a single representative isoform from multiple mRNA isoforms, a series of filtering steps adapted from a previous study[12] was applied. We collected NM sequences to take only mRNAs, eliminating non-coding RNAs. mRNAs mapped on the 24 human chromosomes were included discarding others including 'random' sequences. mRNA isoforms that contain a wrong start or stop codon or whose length is not a multiple of three were discarded. Candidates for nonsense-mediated mRNA decay, whose stop codon is located >50 nts upstream of the last exon-exon junction[13], were also filtered out. Accordingly, we obtained 18,997 representative non-redundant isoforms and used this set of reference mRNAs for subsequent computational analyses.

Selection of miRNAs for overexpression experiments

For a series of experiments to monitor the changes of mRNA expression upon miRNA overexpression on a transcriptome-wide scale, candidate miRNAs were selected by the following steps. Starting from 2,588 total human mature miRNAs in miRbase[14] (Release 21, http://mirbase.org/), we removed miRNAs whose miRBase accession number exceeds 1,000 because many of them are likely to be false positives, resulting in 940 miRNAs.

To select candidate miRNAs for mRNA-seq in the HepG2 cell line, 223 miRNAs that are members of miRNA families broadly conserved across vertebrates were selected[15]. To exclude miRNAs highly expressed in the HepG2 cell line, the top 50 miRNA families with the highest numbers of reads per million mapped reads

(RPM) measured from small RNA-seq (sRNA-seq) were filtered out. Remaining miRNAs were sorted by the evolutionary conservation of the miRNA seed region, which was measured by the sum of PhyloP scores of the seed region obtained from a multiple alignment of 100 vertebrate genomes[16]. In the end, top-ranked 28 miRNAs were used for mRNA-seq. Synthetic miRNA duplexes were designed to make sure the desired strand is dominantly loaded into AGO (**Supplementary Table 3**).

Preprocessing and alignment of sequenced reads

mRNA-seq and small RNA-seq (sRNA-seq) libraries were sequenced using Illumina HiSeq. An AGO2 PAR-CLIP-seq dataset was obtained from a previous study (GSE21578)[17] and PAR-CLIP-seq datasets for HuR and Dnd1 were obtained from previous studies (GSE29943 and PRJNA352073)[18, 19]. Sequenced reads were preprocessed through four steps as listed below. Low sequencing quality regions were trimmed by the custom script in Python, and adapter sequences (Illumina TruSeq RA3) at the 3′ ends were removed by cutadapt-1.2.1[20]. Sequencing artifacts or PCR duplicates were eliminated by fastx_artifact_filter of the FASTX-Toolkit package (hannonlab.cshl.edu/fastx_toolkit/). Reads derived from tRNA or rRNA genes were removed by aligning to the tRNA and rRNA genes using Bowtie2[21].

For mRNA-seq and PAR-CLIP-seq for HuR and Dnd1, preprocessed reads were aligned to the reference human genome (hg38) with TopHat v2.0.14[22] with the following parameters: '`--no-coverage-search --b2-very-sensitive --no-novel-juncs`'. For sRNA-seq, preprocessed reads were aligned to the human mature miRNA sequences (miRBase 21) using Bowtie2 with following parameters: '`-t -k 101 --very-sensitive --norc --score-min C,0,0 --mp 8,8 --np 8`'. Unmapped reads were aligned again using Bowtie2 with a more lenient score cutoff '`-t -k 101 --very-sensitive --norc --score-min C,-8,0 --mp 8,8 --np 8`', which allows a single mismatch during alignment. The sequenced samples are listed in **Supplementary Table 2**.

For AGO2 PAR-CLIP-seq, preprocessing steps and options similar to those of mRNA-seq data were applied except that Illumina Small RNA Adapter sequences, instead of Illumina TruSeq RA3, were used to trim adapter reads as in a previous study[17]. After the adapter trimming step, the sequencing reads were

directly aligned to the human genome (hg38) using TopHat, without artifact filtering and removal of tRNA or rRNA reads.

Transcriptome data processing

We produced a large-scale transcriptome dataset by mRNA-seq in HepG2, HEK293T (Parental, *PCBP2* KO, and *IGF2BP1* KO), and HCT116 cell lines (**Supplementary Table 2**). From the reads aligned to the reference human genome, mRNA expression levels were quantified as the number of reads per kilobase of exon per million mapped reads (RPKM). To reduce experimental noise, we used averaged RPKM value from multiple replicates transfected with the same miRNA. The same process was applied to the control samples (mock-transfected) to obtain mRNA expression profile without the miRNA overexpression. For mRNA expression levels in HEK293T cells, quantile normalization was performed in order to reduce batch effect. Comparing the expression levels between miRNA overexpressed and control data, $\log_2$(mRNA fold change) was calculated. Microarray data from previous studies, which monitored whole-transcriptome response after ectopic introduction of miRNAs or siRNAs in HeLa and other human cancer cell lines, were collected (**Supplementary Table 2**)[23, 24, 25, 26, 27, 28]. For HeLa cell line, a dataset of 74 microarrays after transfecting 100 nM of miRNAs or siRNAs into the cells was obtained from a previous study[23]. The provided list of $\log_2$(mRNA fold change) of each mRNA was used. 102 microarray data after transfecting 10 nM of miRNAs into 12 types of human cancer cell lines were also obtained[24, 25, 26, 27, 28]. The microarray data dataset was produced using Agilent microarray platforms, and the raw data were processed using Bioconductor packages 'agilp', 'GEOquery', 'annotate', and 'limma' in R (http://www.r-project.org). As a result, mRNA expression levels were obtained and $\log_2$(mRNA fold change) after miRNA transfection was calculated from the raw signals. For both microarray and mRNA-seq datasets, $\log_2$(mRNA fold change) was normalized with respect to the AU content of ORF sequence by LOWESS filtering to correct for the potential AU bias[29]. '`lowess()`' function in Biopython package (https://biopython.org/) was used for the normalization with the following parameters: f=0.05, iteration=1.

Quality control of miRNA overexpression experiments

To screen experiments where miRNA duplexes were properly transfected into cells, we applied a few criteria for quality control and selected a subset of them. First, we ruled out low-quality experiments, where there is no significant repression of targets (defined as 3′UTRs with ≥1 7, 8mer MTSs) compared to non-targets (defined as 3′UTRs without any MTS, 'No site' group). Second, for each site type of 8mer, 7mer-m8, 7mer-A1, and 6mer, if 3′UTRs that include an MTS of the site type show significant upregulation compared to 'No site' group, the experiment was discarded. Third, if the experiment did not exhibit the known hierarchy of miRNA targeting (MT) efficacies among MTS types (8mer>7mer>6mer)[30], the experiment was also excluded. For the dataset of HCT116 cell line which depleted miRNA expression by *DICER* or *DROSHA* KO (GSE77989) [31], the opposite direction of the above patterns was examined. Lastly, we measured the distribution of $\log_2$(mRNA fold change) of 'No site' group and discarded the experiment, if <90% of the data points are included in the expected range between −1.0 and 1.0, as it was considered to be noisy.

As a result, 36 out of 45 mRNA-seq experiments in HepG2 cell line, both of 2 mRNA-seq experiments in HCT116 cell line, 59 out of the 74 microarray experiments in HeLa cell line, and 75 out of the 102 microarray experiments in other various human cancer cell lines were selected. From the samples obtained from HEK293T (Parental, *PCBP2* KO, and *IGF2BP1* KO) mRNA-seq, we selected pairs of samples if significant repression of target mRNAs was observed for both parental cells and RBP KO cells, applying the aforementioned criteria of comparison between 'No site' group and the target mRNAs. As a result, 8 and 9 pairs of samples were selected in *IGF2BP1* KO and *PCBP2* KO cells, respectively. Top 50% of the most highly expressed mRNAs from each mRNA-seq or microarray dataset were used for subsequent analyses. Wilcoxon's rank-sum test of 'SciPy' package in Python was used and a P value of <0.01 was regarded as statistically significant.

Correlation analysis of RBP-binding sites between the different cell lines

To examine whether the RBSs are robustly preserved between HepG2 and K562 cell lines, we calculated the correlation between the RBSs of those RBPs profiled in both cell lines. 3′UTRs with top 25% of the most highly expressed in both cell lines were collected. Each 3′UTR was partitioned into 10 nt windows and then each window was assigned to be 'RBS presence' if any of the ten nucleotides includes an RBS and 'RBS

absence' otherwise. The correlation of the RBS presence or absence between the two cell lines were calculated by using Yule's *Y* value[32], after converting 'RBS presence' and 'RBS absence' windows into 1 and 0, respectively. Those RBPs that belong to top 25% of the most highly expressed genes in both cell lines were used and the correlation was examined for lenient (**Supplementary Fig. 1d**) or stringent (**Supplementary Fig. 1e**) RBSs. The correlation *P* value was calculated by the χ^2 test for a 2×2 contingency table using 'SciPy' package in Python.

Association analysis between the number of RBPs binding close to the MTS and MT efficacy

We conducted a series of analyses to investigate the collective effect of RBP binding on MT efficacy in unbiased manner (**Fig. 3a-c and Supplementary Figs. 2-4**). From the transcriptome data of HepG2, HeLa, and other human cancer cell lines, mRNAs that contain a single 7, 8mer MTS in 3′UTR and that are within top 50% in expression level were collected. For each MTS, the number of bound RBPs located within 50 nts from the MTS was counted using CITSs. The MTSs were then separated into five groups by the number of bound RBPs while controlling other confounding features of MT, as described above. Log_2(mRNA fold change) or other confounding features were compared among the groups using Wilcoxon's rank-sum test of 'SciPy' package in Python (**Fig. 3a and Supplementary Fig. 2**).

To test whether the detected collective effect of RBPs on MT is determined by specific RBPs, we compared the efficacy of MTSs with similar numbers of bound RBPs but with different RBP compositions. First, MTSs with the number of bound RBPs between 1 and 10 were collected from HepG2 dataset. For each MTS, the multi-dimensional binding information of the RBPs that have one or more RBSs within 50 nt flanking regions from the MTS was reduced into three-dimensional information by the tSNE[33] method (**Fig. 3b**, right panel). A shorter distance between two MTSs within the reduced dimension represents a higher similarity of RBP-binding composition. Using *k*-means clustering, MTSs were divided into two subgroups with close distances. Therefore, MTSs with similar RBP-binding composition are grouped and the RBP-binding composition of a group is distinct from the other group. Then, pairs of MTSs that have similar values of the number of bound RBPs and other confounding features of MT were selected from the two MTS subgroups as described above, and the log_2(mRNA fold change) of the MTS subgroups were compared with each other (**Fig. 3c**, right panel). An identical analysis was repeated for the MTSs with different ranges for the number of bound

RBPs, or datasets for HeLa and other human cancer cell lines (**Supplementary Figs. 3 and 4**). tSNE and *k*-means clustering was conducted using 'scikit-learn' package in Python and Wilcoxon's rank-sum test of 'SciPy' package in Python was used for statistical test.

Examination on motif-specific RBP-binding sites

To examine whether the observed association between the number of RBPs bound and MT efficacy is influenced by motif specificity of RBP binding, we divided RBSs provided by the ENCODE eCLIP-seq dataset into motif-specific or motif-unspecific binding sites. From the mCross database[6], sequence motifs for available 99 RBPs were obtained. For each RBP, each of their RBSs was classified to be motif-specific if the RBS contains one or more mCross motif sequences of the RBP. Accordingly, 10% of RBSs were considered to be motif-specific on average and used for subsequent analyses. For each subset of RBSs separated by their motif-specificity, association analyses shown in **Fig. 3a** were performed (**Supplementary Fig. 2e**).

AGO2 PAR-CLIP-seq analysis

To test the association between the increase of AGO occupancy upon miRNA overexpression and RBP binding, we adopted AGO2 PAR-CLIP-seq dataset generated in HEK293 cells[17]. The dataset is comprised of AGO2 PAR-CLIP-seq samples in response to ectopically introduced miR-7, miR-124, and mock conditions. The raw sequencing data were preprocessed and aligned as described above. For AGO2 PAR-CLIP-seq samples of normal and miR-124-transfected conditions, AGO2 occupancy was measured as the number of reads per million mapped reads (RPM) within 50 nt flanking regions from each of 7, 8mer MTSs of miR-124 in 3′UTRs. Log_2(fold change) of AGO2 occupancy for each MTS was calculated by comparing RPM values of the normal sample and that of the miR-124-transfected sample, while applying 1.0 as a pseudo count for the calculation of the $\log_2$(fold change). The magnitude of RBP-binding signal within 50 nt flanking regions from each MTS was measured by using CITSs. The MTSs were then separated into three subgroups by the magnitude of their RBP-binding signal, and the $\log_2$(fold changes) of AGO2 occupancy were compared between subgroups using Wilcoxon's rank-sum test of 'SciPy' package in Python. An identical analysis was performed for ectopically introduced miR-7 (**Fig. 5a**).

Prediction of local secondary structures

We calculated the changes of secondary structures upon RBP-unbound or RBP-bound states (**Fig. 5b and Supplementary Fig. 5a**). The minimum free energy and secondary structures of the flanking regions of 50 nts upstream and downstream of MTS were predicted by RNAfold (v.2.4.13) in Vienna RNA package[34, 35]. Four different states of structures (ΔG_0, ΔG_{miR}, ΔG_{RBP} and $\Delta G_{RBP+miR}$) depending on the miRNA-loaded AGO or RBP binding to the 3′UTR were predicted by giving the constraint option (RNAfold -C) and constraining MTSs and RBSs to be single-stranded states. The input sequence and four types of structural constraints were used for each state as shown in the following examples.

```
NNNNNNNNNN...NNNNNNNNNNN...NNNNNNNNNNN  Sequence (107 or 108 nts)
......................................  (ΔG0) No constraint
...............xxxxxxx................  (ΔGmiR) Constraining only to the MTS
.xxxxx..........................xxxxxxxxx.  (ΔGRBP) Constraining only to the RBS
.xxxxx.........xxxxxxx......xxxxxxxxx.  (ΔGRBP+miR) Constraining to both MTS and RBS
```

where the underline represents an MTS and the 'x' indicates residues that are supposed be single-stranded. To measure the structural change of 3′UTRs in RBP-unbound state, the differences of minimum free energies before and after miRNA-loaded AGO binding were calculated by $\Delta G_{miR} - \Delta G_0$ (**Fig. 5b**). For RBP-bound state, we used CITSs as the RBP-binding signal and the structural change was calculated by $\Delta G_{RBP+miR} - \Delta G_{RBP}$ (**Fig. 5b**). Calculated ($\Delta G_{miR} - \Delta G_0$) and ($\Delta G_{RBP+miR} - \Delta G_{RBP}$) values were used for the analysis in **Supplementary Fig. 5a**.

Comparison of regression models

For each of 150 RBPs in the eCLIP-seq dataset, MLR models of simple ternary interaction of miRNA, AGO, and target mRNA, and our proposed model were fitted to the observed fold changes of the target mRNAs for datasets of HepG2, HeLa, and other human cancer cell lines. We tested whether our proposed model provides improved interpretation of MT efficacy compared to the simple ternary interaction model. Using one-sided Steiger's *z* test[36], correlations between the observed mRNA fold changes and predicted fold

changes by the MLR models were compared, illustrating which of the two models better fits to the observed data. Python implementation of the 'cocor' package[37] in R was used for one-sided Steiger's *z* test. For estimated *P* values on each RBP, the false discovery rate (FDR) correction was conducted (**Supplementary Fig. 5a**) and *q* values under 0.01 were regarded to be statistically significant.

Determination of RBP functions

To confirm whether the enhanced MT efficacy for MTSs that overlap with RBSs is dependent on the functions of RBPs, we separated each RBP into one of two categories by its estimated function: whether it stabilizes or destabilizes mRNA targets (**Fig. 6c**). We used the ENCODE mRNA-seq data that monitored the whole-transcriptome response to RBP depletion by shRNA treatment. Experimental data for 68 RBPs in HepG2 cell line and 80 RBPs in K562 cell line were collected. To confirm the depletion of the targeted RBP, we compared control and knockdown samples and identified RBPs that exhibited >50% depletion. As a result, we selected 26 out of 68 RBPs in HepG2 cell line and 53 out of 80 RBPs in K562 cell line.

For each selected RBP, top 50% of the most highly expressed mRNAs in control sample were used and the on-target and off-targets of the overexpressed shRNA were excluded. mRNAs that contain ≥1 6-8mer MTSs in their 3′UTRs or ≥1 7, 8mer MTSs in their ORFs were regarded as shRNA off-targets. We classified the remaining mRNAs into either RBP targets, where the mRNA contains ≥1 3′UTR RBS, or non-targets otherwise. Wilcoxon's rank-sum test in Python was used to examine whether the $\log_2$(fold changes) of RBP targets were significantly different from that of the non-targets. As a result, we determined that 17 and 13 RBPs function as destabilizers and stabilizers, respectively, with the FDR-corrected *q* value cutoff of 0.05.

Examination on RBPs reported to suppress or enhance MT

To assess the MT regulatory impact of binding of RBPs that have been previously reported as MT suppressors or enhancers, we collected the list of MT suppressor and enhancer RBPs through a literature survey. Accordingly, IGF2BP1-3, Dnd1, HuR, and PTBP1 were categorized as previously reported MT suppressors[38, 39, 40, 41], while Pumilio, PCBP2, FMR1, TARDBP, HuR, and PTBP1 as enhancers[2, 41, 42, 43, 44, 45]. Among these RBPs, ENCODE eCLIP-seq data were not available for Dnd1 and HuR proteins and thus

we obtained additional PAR-CLIP-seq data for these two RBPs[18, 19]. eCLIP-seq and PAR-CLIP-seq data for the MT enhancer and suppressor RBPs were used for analysis in **Fig. 6g**.

To process PAR-CLIP-seq and mRNA-seq data for Dnd1 and HuR, we preprocessed the data as described above. Read coverage across the human genome was calculated for both PAR-CLIP-seq and mRNA-seq data and the coverage of PAR-CLIP-seq data was normalized such that the total number of mapped reads is identical to that of mRNA-seq data. Using a dynamic programming algorithm, genomic regions where the PAR-CLIP-seq reads were highly enriched compared to the mRNA-seq reads were collected as RBS candidates. For each of the collected RBS candidates, a 2×2 contingency table was constructed by examining the normalized number of reads for an RBS candidate and its 10,000 nt flanking region as a control for PAR-CLIP-seq and mRNA-seq data. χ^2 test was conducted for the contingency table and the *P* values for RBS candidates were corrected for multiple testing by the FDR-correction. The candidate regions whose *q* values are less than 0.01 were determined as RBSs. Using the RBSs of MT suppressor or enhancer RBPs, the analysis shown in **Fig. 1d** were conducted (**Fig. 6g**).

Association analysis between RBPs and MT efficacy for subgroups of MTSs and RBPs

Analyses identical to **Fig. 1d** were conducted except for focusing on the subpopulation of mRNAs, or on the subpopulation of RBPs (**Figs. 4a, 6a-g, and Supplementary Fig. 5b**). For subset of mRNAs containing a single MTS with identical MTS type (8mer, 7mer-m8, 7mer-A1, or 6mer) in their 3′UTRs, the analysis shown in **Fig. 1d** was iterated. Similarly, to evaluate ORF MTSs, a similar analysis was iterated for mRNAs that do not contain any 6-8mer MTS in their 3′UTRs and that contain a single 8mer MTS in their ORFs (**Supplementary Fig. 5c**). When dissecting the effect of RBPs by their various properties, a similar analysis was conducted by calculating the distances between MTSs and the RBSs for the subset of RBPs. Function of RBPs regarding the mRNA stability was assigned by our analysis as described above (**Fig. 6c**). Based on the gene ontology annotation [46], RBPs with a helicase domain, single-stranded RNA binding activity, or double-stranded RNA (dsRNA) binding activity were collected (**Fig. 6d, e**). A list of RBPs that directly interact with AGO or TNRC6 was obtained from a previous study[47] (**Fig. 4a**). Cytoplasmic fraction of each RBP was determined by analysis used for **Supplementary Fig. 1b** (**Fig. 6f**), and RBPs that act as MT enhancers or MT suppressors were collected by literature survey as explained above (**Fig. 6g**). To more

clearly dissect the impact of dsRNA-binding proteins (dsRBPs) on MT efficacy, we calculated the fraction of their RBS nucleotide regions overlapping with RBSs of the other RBPs. For subgroups of dsRBPs with low or high overlapping fraction, analyses of **Fig. 1d** were performed (**Supplementary Fig. 6**). Similar analyses were also performed for the RBPs with lower cytoplasmic fraction (**Supplementary Fig. 1b**).

For the analyses in **Fig. 3a**, procedures similar to **Fig. 1d** were performed with the following differences. MTSs were subsampled and separated into five subgroups with a different number of bound RBPs within 50 nt flanking regions of the MTS, from the absence of an RBS (None) to a large number of RBSs, while controlling the confounding features for MT. For HepG2 and HeLa dataset, expression level of mRNAs was further controlled to have comparable RPKM values among subgroups using mRNA-seq data of corresponding cell lines. To more thoroughly investigate the MT regulatory impact mediated by of a small number of bound RBPs, a modified version of analyses was conducted (**Supplementary Fig. 2c**): instead of separating MTSs into five subgroups, MTSs were divided into four subgroups and in the second bin we chose MTSs that include five or less number of bound RBPs. In **Fig. 3c**, the MTSs from two subgroups were further subsampled to have statistically indistinguishable confounding features and the number of bound RBPs (**Supplementary Fig. 3c**).

Design of 3′UTR constructs with mutated MTSs or RBSs

Mutated sequences of the miRNA target 3′UTRs were produced to detect the effect of RBP binding on MT efficacy (**Fig. 8**). Mutated MTSs was generated by randomly shuffling the sequence of an MTS. After removing those shuffled MTSs that have a different number of CpG dinucleotides compared to the original MTS or that are matching to the seed site of evolutionary conserved miRNAs[15], one of the remaining shuffled MTSs was randomly chosen as the mutated MTS candidate.

For mutated RBSs, we used precise locations of RBSs determined from the CITS counts. We selected two nucleotide positions of the strongest CITS signals within the 3′UTR fragment that include the MTS and its 50 nt flanking regions in both 5′ and 3′ directions and termed these two nucleotide positions as CITS peaks. For the first CITS peak, we chose a 5mer sequence whose center is on the CITS peak and randomly shuffled the 5mer. To make sure the shuffled 5mer does not change the local secondary structure of the original sequence, the minimum free energy, ΔG_{MUT} was calculated by the RNAfold software[34, 35] and

compared with that of the original sequence, ΔG_{WT}. Among the randomly shuffled 5mers, a 5mer that has the smallest difference ($\Delta G_{MUT} - \Delta G_{WT}$) was selected. The same selection process was repeated for the second CITS peak and a mutated RBS was made by inserting these two randomly shuffled 5mers.

Generation of RBP knockout cell lines

RBP knockout cell lines were established using a CRISPR-Cas9 system as previously described[48]. To prepare for guide RNA expression constructs, guide oligos were synthesized (Bioneer) and were inserted into pSpCas9(BB)-2A-Puro vector expressing Cas9. The guide oligos of each RBP are as follow: *PCBP2* guide, 5′-GACACCGGTGTGATTGAAGG-3′; *IGF2BP1* guide, 5′-GAGCGTGACCCCCGCGGACT-3′. For generation of knockout cell lines, HEK293T were seeded in a 6-well plate. One day later, seeded HEK293T were transfected with 3 μg of the plasmids using Lipofectamine 2000 (Invitrogen) and incubated for 48 hours. After incubation, cells were treated with 3 μg/mL Puromycin (Gibco). By western blot analysis, the knockout clones were selected. Each representative KO clone #1 was genotyped by PCR to validate the indel mutations (**Supplementary Fig. 7c and Supplementary Table 4**).

For the rescue experiments, *PCBP2* and *IGF2BP1* genes were amplified by PCR with specific primers containing its coding sequences and inserted into pcDNA3.1 vector containing FLAG-tag (**Supplementary Table 4**). Each construct was transfected to *PCBP2* or *IGF2BP1* knockout cell lines and incubated for 24 hours.

Western blot analysis and co-immunoprecipitation

For western blot analysis after generation of RBP knockout cell lines, whole cell extracts were prepared by RIPA buffer (Thermo Scientific) with proteinase inhibitor cocktail (Roche).

For western blot analysis after RNA immunoprecipitation (IP), HEK293T WT and RBP KO (*PCBP2* and *IGF2BP1*) cells were seeded in 150-mm plates at ~60% confluency. Then, transfection, lysis, and immunoprecipitation were performed by following the protocol below. Image was obtained using FusionCapt Advances Solo 2 v. 17.01.

For co-immunoprecipitation assay, modified version of protocol from a previous study was used[49]. Specifically, HEK293T cells were transfected with FLAG-hAGO2 plasmid and incubated for 24 hours. Then,

cells were lysed using NET-2 buffer[49]. The lysates were either treated with 4μg of RNase A or not treated and were incubated for 20min at 37°C. Then, the lysates were incubated with anti-FLAG M2 affinity gel (Sigma) at 4°C for 2 hours. After immunoprecipitation, the immunoprecipitated sample was washed 5 times with NET-2 buffer. Co-immunoprecipitated proteins were visualized by western blot. The image was obtained using Amersham Imager 600 (Cytiva).

The lysates and IPed samples were resolved on 10% SDS-PAGE and transferred to PVDF membrane (EMD Milipore). The membrane was incubated with primary antibodies to rabbit anti-PCBP2 (MBL), rabbit anti-IGF2BP1 (MBL), rabbit anti-LARP4 (Bethyl Lab), rabbit anti-PABP1 (abcam), mouse anti-FLAG (Sigma), rabbit anti-AGO2 (Cell Signaling), and rabbit anti-GAPDH (Cell Signaling). Secondary antibodies were anti-rabbit IgG and anti-mouse IgG antibody (BIORAD).

Selection of pairs of miRNA and mRNA targets for reporter assay

For luciferase reporter assays in HepG2 and HEK293T cell lines in **Fig. 8a, b**, same filtering processes were applied which were used for selection of miRNAs for overexpression experiments. To select 3′UTRs for luciferase reporter assay, mRNAs with 3′UTR length of <600 nts were chosen since long 3′UTRs tend to be unresponsive to miRNAs[50]. Then, mRNAs that are moderately expressed (top 2-40%) based on the mRNA expression level in HepG2 and HEK293T cell lines, that contain a single 7, 8mer 3′UTR MTS, and that contain RBSs within 50 nts from the MTS were selected. mRNAs with MTSs located close to the stop codon (<20 nts) were discarded[30]. For the reporter assay in **Fig. 8a**, instead of focusing on binding sites of a specific RBP, we chose 3′UTRs that contain one or more RBSs that various RBPs would bind, to observe the general impact of RBP binding to the MT efficacy. Among 3′UTRs that contain a single 7, 8mer MTS in 3′UTR and RBSs within 50 nts from the MTS, 3′UTRs with strong RBP-binding signals near MTSs were selected. For **Fig. 8b**, top 3′UTRs with CITS profile mainly dominated by PCBP2 and IGF2BP1 for *PCBP2* KO and *IGF2BP1* KO rescue reporter assay respectively were selected to monitor the effect of absence and regaining of PCBP2 or IGF2BP1. The same 3′UTRs were used as the targets for AGO2-IP qPCR assay in **Fig. 7f, g**.

For each of all possible candidate pairs of the miRNAs and the mRNAs, a context score of the MTS was predicted as previously described[12], which is similar to the context+ score of TargetScan6[23] (http://www.targetscan.org/vert_60/) and the pairs with the context score <−0.2 were selected.

Transcriptome-wide analysis for global impact of RBPs on MT

For comprehensive observation of the regulatory effect of RBPs on MT, we performed mRNA-seq and measured the transcriptome response after the transfection of miRNAs on HEK293T parental cells and *IGF2BP1* or *PCBP2* knockout (KO) cells, respectively. For each of these three cells, mRNA-seq was performed after miRNA or mock transfection. The transfected miRNAs are listed in **Supplementary Table 2**.

Expression levels of mRNAs were quantified as the number of reads per kilobase of exon per million mapped reads (RPKM). To reduce the noise, mock-transfected samples for each cell type were merged by averaging RPKMs. Comparing the expression levels between miRNA overexpressed and control data, $\log_2$(mRNA fold change) was calculated and was normalized as explained in mRNA-seq data processing section. To measure the change of MT efficacy upon RBP deletion, the values were obtained by subtracting $\log_2$(mRNA fold change) of parental cells from that of RBP KO cells for each mRNA. A positive value indicates the de-repression of target mRNAs, implying that KO of the RBP mediates the decrease of MT efficacy. Top 50% mRNAs of the most highly expressed in mock-transfected parental cells were used for the analysis. Target mRNAs that contain a single 7, 8mer MTS in their 3′UTRs were selected if the distance between the MTS and the nearest RBS, denoted as $d_{\text{MTS-RBS}}$, is short ($d_{\text{MTS-RBS}}$ <100) or long ($d_{\text{MTS-RBS}}$ ≥100). For a fair comparison between the short and long $d_{\text{MTS-RBS}}$ subgroups, confounding features of MT were controlled as described above. Calculated changes of MT efficacy upon removal of RBP were then compared between the controlled subgroups using Kolmogorov-Smirnov test of 'SciPy' package in Python (**Fig. 8c and Supplementary Fig. 8a, b**). To confirm that the observed change in MT efficacy upon RBP deletion is not biased by the own function of the RBP on mRNA abundance, mRNAs without any MTS in the 3′UTR ('No-site' group) were collected after control of mRNA expression level and 3′UTR length, and their de-repression values were compared to the values of miRNA targets.

Assessment of global impact of RBPs on MT

Fraction of MTSs that have RBSs in their close proximity was calculated to assess the global impact of RBP binding on MT in the human genome (**Fig. 9a**). 7, 8mer MTSs for 108 miRNA families broadly conserved across vertebrates were collected from top 50% of the most highly expressed mRNAs for HepG2 and K562 cell lines. The fraction of the MTSs that contain RBSs within 50 or 100 nt flanking regions from the MTS was calculated using the CITS counts. Given that there exist >1,500 human RBPs[51], we extrapolated the analysis to a larger number of considered RBPs. To estimate the expected fraction, RBPs were randomly subsampled, and the fraction of MTSs located close to RBSs was calculated for the subsampled RBPs. The calculated fractions were fit to the logarithmic curve using 'SciPy' package in Python. The mean PhyloP score[16] within nucleotides of the MTSs was calculated, and the analysis was repeated for the conserved MTSs (PhyloP>0.0) and highly conserved MTSs (PhyloP>2.0) and for HepG2 and K562 cell lines.

Examination on co-occurrence of MTSs and RBSs

From GTEx database[52], expression levels of mRNAs and miRNAs, provided as the number of transcripts per million (TPM), were obtained for 54 human tissue types. To test the co-occurrence between MTSs of evolutionarily conserved miRNAs and RBSs, the enrichment of RBP binding near the MTSs of conserved and highly expressed miRNAs were compared to that of control miRNAs. For each tissue, a miRNA that is most highly expressed and also broadly conserved across vertebrates[15] was selected. Then, as a negative control, a miRNA with the lowest expression level and with identical nucleotide composition and CpG count in the seed region to that of the conserved miRNA was selected. Those selected pair of miRNAs were excluded from the list of miRNAs, and the selection process above was iterated until 15 miRNA pairs were selected.

For each tissue, top 50% of the most highly expressed mRNAs and top 10% of the most highly expressed RBPs were used for analysis. 7, 8mer MTSs in 3′UTRs were collected for each of miRNAs, and the magnitudes of RBP-binding signal were calculated by counting CITSs of the highly expressed RBPs within 50 nt flanking region of MTSs. For a fair comparison of RBP-binding signals between MTSs of conserved miRNAs and their control miRNAs, MTSs of each pair of miRNAs were controlled to have similar values of

TPM, PhyloP scores of MTS, and PhyloP scores of 50 nt flanking region of MTS (**Supplementary Fig. 8c**). After controlling for potentially confounding features, the magnitudes of RBP-binding signal of MTSs were compared between conserved miRNAs and their controls using Wilcoxon's rank-sum test of 'SciPy' package in Python. Enrichment or depletion of RBP-binding signals was determined by *q* values corrected by the false discovery rate for each tissue (**Fig. 9b**).

References

1. Rouskin S, Zubradt M, Washietl S, Kellis M, Weissman JS. Genome-wide probing of RNA structure reveals active unfolding of mRNA structures in vivo. *Nature* **505**, 701-705 (2014).

2. Kedde M, van Kouwenhove M, Zwart W, Oude Vrielink JA, Elkon R, Agami R. A Pumilio-induced RNA structure switch in p27-3' UTR controls miR-221 and miR-222 accessibility. *Nat Cell Biol* **12**, 1014-1020 (2010).

3. Lorsch JR. RNA chaperones exist and DEAD box proteins get a life. *Cell* **109**, 797-800 (2002).

4. Rajkowitsch L*, et al.* RNA chaperones, RNA annealers and RNA helicases. *RNA Biol* **4**, 118-130 (2007).

5. Roth BM, Ishimaru D, Hennig M. The Core Microprocessor Component DiGeorge Syndrome Critical Region 8 (DGCR8) Is a Nonspecific RNA-binding Protein. *Journal of Biological Chemistry* **288**, 26785-26799 (2013).

6. Feng HJ*, et al.* Modeling RNA-Binding Protein Specificity In Vivo by Precisely Registering Protein-RNA Crosslink Sites. *Molecular Cell* **74**, 1189-+ (2019).

7. Weyn-Vanhentenryck SM*, et al.* HITS-CLIP and Integrative Modeling Define the Rbfox Splicing-Regulatory Network Linked to Brain Development and Autism. *Cell Reports* **6**, 1139-1152 (2014).

8. Sundararaman B*, et al.* Resources for the Comprehensive Discovery of Functional RNA Elements. *Mol Cell* **61**, 903-913 (2016).

9. Abràmoff MD, Magalhães PJ, Ram SJ. Image processing with ImageJ. *Biophotonics international* **11**, 36-42 (2004).

10. Guo H, Ingolia NT, Weissman JS, Bartel DP. Mammalian microRNAs predominantly act to decrease target mRNA levels. *Nature* **466**, 835-840 (2010).

11. Pruitt KD, Tatusova T, Maglott DR. NCBI reference sequences (RefSeq): a curated non-redundant sequence database of genomes, transcripts and proteins. *Nucleic Acids Res* **35**, D61-65 (2007).

12. Kim D*, et al.* General rules for functional microRNA targeting. *Nat Genet* **48**, 1517-1526 (2016).

13. Nagy E, Maquat LE. A rule for termination-codon position within intron-containing genes: when nonsense affects RNA abundance. *Trends in biochemical sciences* **23**, 198-199 (1998).

14. Kozomara A, Griffiths-Jones S. miRBase: annotating high confidence microRNAs using deep sequencing data. *Nucleic Acids Res* **42**, D68-73 (2014).

15. Friedman RC, Farh KK, Burge CB, Bartel DP. Most mammalian mRNAs are conserved targets of microRNAs. *Genome Res* **19**, 92-105 (2009).

16. Pollard KS, Hubisz MJ, Rosenbloom KR, Siepel A. Detection of nonneutral substitution rates on mammalian phylogenies. *Genome Res* **20**, 110-121 (2010).

17. Hafner M*, et al.* Transcriptome-wide identification of RNA-binding protein and microRNA target sites by PAR-CLIP. *Cell* **141**, 129-141 (2010).

18. Yamaji M*, et al.* DND1 maintains germline stem cells via recruitment of the CCR4-NOT complex to target mRNAs. *Nature* **543**, 568-572 (2017).

19. Lebedeva S*, et al.* Transcriptome-wide analysis of regulatory interactions of the RNA-binding protein HuR. *Mol Cell* **43**, 340-352 (2011).

20. Martin M. Cutadapt removes adapter sequences from high-throughput sequencing reads. *2011* **17**, 3 (2011).

21. Langmead B, Salzberg SL. Fast gapped-read alignment with Bowtie 2. *Nat Methods* **9**, 357-359 (2012).

22. Trapnell C, Pachter L, Salzberg SL. TopHat: discovering splice junctions with RNA-Seq. *Bioinformatics* **25**, 1105-1111 (2009).

23. Garcia DM, Baek D, Shin C, Bell GW, Grimson A, Bartel DP. Weak seed-pairing stability and high target-site abundance decrease the proficiency of lsy-6 and other microRNAs. *Nat Struct Mol Biol* **18**, 1139-1146 (2011).

24. Yamada Y*, et al.* Regulation of antitumor miR-144-5p targets oncogenes: Direct regulation of syndecan-3 and its clinical significance. *Cancer Sci* **109**, 2919-2936 (2018).

25. Yamada Y*, et al.* Passenger strand of miR-145-3p acts as a tumor-suppressor by targeting MYO1B in head and neck squamous cell carcinoma. *Int J Oncol* **52**, 166-178 (2018).

26. Yamada Y*, et al.* Molecular pathogenesis of renal cell carcinoma: Impact of the anti-tumor miR-29 family on gene regulation. *Int J Urol* **25**, 953-965 (2018).

27. Kumamoto T*, et al.* Regulation of TPD52 by antitumor microRNA-218 suppresses cancer cell migration and invasion in lung squamous cell carcinoma. *Int J Oncol* **49**, 1870-1880 (2016).

28. Idichi T*, et al.* Molecular pathogenesis of pancreatic ductal adenocarcinoma: Impact of passenger strand of pre-miR-148a on gene regulation. *Cancer Sci* **109**, 2013-2026 (2018).

29. Elkon R, Agami R. Removal of AU bias from microarray mRNA expression data enhances computational identification of active microRNAs. *PLoS Comput Biol* **4**, e1000189 (2008).

30. Grimson A, Farh KK, Johnston WK, Garrett-Engele P, Lim LP, Bartel DP. MicroRNA targeting specificity in mammals: determinants beyond seed pairing. *Mol Cell* **27**, 91-105 (2007).

31. Kim YK, Kim B, Kim VN. Re-evaluation of the roles of DROSHA, Export in 5, and DICER in microRNA biogenesis. *Proc Natl Acad Sci U S A* **113**, E1881-1889 (2016).

32. Yule GU. On the methods of measuring association between two attributes. *J R Stat Soc* **75**, 579-652 (1912).

33. van der Maaten L, Hinton G. Visualizing Data using t-SNE. *J Mach Learn Res* **9**, 2579-2605 (2008).

34. Lorenz R*, et al.* ViennaRNA Package 2.0. *Algorithms Mol Biol* **6**, 26 (2011).

35. Lorenz R, Hofacker IL, Stadler PF. RNA folding with hard and soft constraints. *Algorithms Mol Biol* **11**, 8 (2016).

36. Steiger JH. Tests for comparing elements of a correlation matrix. *Psychological Bulletin* **87**, 245-251 (1980).

37. Diedenhofen B, Musch J. cocor: A comprehensive solution for the statistical comparison of correlations. *PloS one* **10**, e0121945 (2015).

38. Degrauwe N*, et al.* The RNA Binding Protein IMP2 Preserves Glioblastoma Stem Cells by Preventing let-7 Target Gene Silencing. *Cell Rep* **15**, 1634-1647 (2016).

39. Kedde M*, et al.* RNA-binding protein Dnd1 inhibits microRNA access to target mRNA. *Cell* **131**, 1273-1286 (2007).

40. Ahuja D, Goyal A, Ray PS. Interplay between RNA-binding protein HuR and microRNA-125b regulates p53 mRNA translation in response to genotoxic stress. *RNA Biol* **13**, 1152-1165 (2016).

41. Xue Y*, et al.* Direct conversion of fibroblasts to neurons by reprogramming PTB-regulated microRNA circuits. *Cell* **152**, 82-96 (2013).

42. Lin X*, et al.* Interplay between PCBP2 and miRNA modulates ARHGDIA expression and function in glioma migration and invasion. *Oncotarget* **7**, 19483-19498 (2016).

43. Kim HH, Kuwano Y, Srikantan S, Lee EK, Martindale JL, Gorospe M. HuR recruits let-7/RISC to repress c-Myc expression. *Genes Dev* **23**, 1743-1748 (2009).

44. Edbauer D*, et al.* Regulation of synaptic structure and function by FMRP-associated microRNAs miR-125b and miR-132. *Neuron* **65**, 373-384 (2010).

45. Fan Z, Chen X, Chen R. Transcriptome-wide analysis of TDP-43 binding small RNAs identifies miR-NID1 (miR-8485), a novel miRNA that represses NRXN1 expression. *Genomics* **103**, 76-82 (2014).

46. Gene Ontology C. Gene Ontology Consortium: going forward. *Nucleic Acids Res* **43**, D1049-1056 (2015).

47. Landthaler M*, et al.* Molecular characterization of human Argonaute-containing ribonucleoprotein complexes and their bound target mRNAs. *RNA* **14**, 2580-2596 (2008).

48. Ran FA, Hsu PD, Wright J, Agarwala V, Scott DA, Zhang F. Genome engineering using the CRISPR-Cas9 system. *Nature protocols* **8**, 2281 (2013).

49. Park J*, et al.* Misfolded polypeptides are selectively recognized and transported toward aggresomes by a CED complex. *Nat Commun* **8**, 15730 (2017).

50. Kim D, Kim J, Baek D. Global and local competition between exogenously introduced microRNAs and endogenously expressed microRNAs. *Mol Cells* **37**, 412-417 (2014).

51. Gerstberger S, Hafner M, Tuschl T. A census of human RNA-binding proteins. *Nature Reviews Genetics* **15**, 829 (2014).

52. Lonsdale J*, et al.* The Genotype-Tissue Expression (GTEx) project. *Nature Genetics* **45**, 580-585 (2013).
