# Supplementary material for: The regulatory impact of RNA-binding proteins on microRNA targeting
Source: Nat Commun. 2021 Aug 20;12:5057. doi: 10.1038/s41467-021-25078-5 (PMC8379221; doi:10.1038/s41467-021-25078-5)
Supplement: Supplementary file 3 — Reporting Summary [file 41467_2021_25078_MOESM3_ESM.pdf]

nature research

Corresponding author(s): Daehyun Baek

Last updated by author(s): Jul 9, 2021

Reporting Summary

Nature Research wishes to improve the reproducibility of the work that we publish. This form provides structure for consistency and transparency in reporting. For further information on Nature Research policies, see our Editorial Policies and the Editorial Policy Checklist.

Statistics

For all statistical analyses, confirm that the following items are present in the figure legend, table legend, main text, or Methods section.

n/a	Confirmed	
☐	☒	The exact sample size (*n*) for each experimental group/condition, given as a discrete number and unit of measurement
☐	☒	A statement on whether measurements were taken from distinct samples or whether the same sample was measured repeatedly
☐	☒	The statistical test(s) used AND whether they are one- or two-sided<br>*Only common tests should be described solely by name; describe more complex techniques in the Methods section.*
☐	☒	A description of all covariates tested
☐	☒	A description of any assumptions or corrections, such as tests of normality and adjustment for multiple comparisons
☐	☒	A full description of the statistical parameters including central tendency (e.g. means) or other basic estimates (e.g. regression coefficient) AND variation (e.g. standard deviation) or associated estimates of uncertainty (e.g. confidence intervals)
☐	☒	For null hypothesis testing, the test statistic (e.g. F, t, r) with confidence intervals, effect sizes, degrees of freedom and P value noted<br>*Give P values as exact values whenever suitable.*
☒	☐	For Bayesian analysis, information on the choice of priors and Markov chain Monte Carlo settings
☒	☐	For hierarchical and complex designs, identification of the appropriate level for tests and full reporting of outcomes
☒	☐	Estimates of effect sizes (e.g. Cohen's *d*, Pearson's *r*), indicating how they were calculated

Our web collection on statistics for biologists contains articles on many of the points above.

Software and code

Policy information about availability of computer code

Data collection	Roche Lightcycler480 software Ver. 1.5.1<br>GloMax 96 Microplate Luminometer Software Ver.1.9.2<br>FusionCapt Advance Solo 2 Software Ver. 17.01<br>Amersham Imager 600 Ver. 1.2.0
Data analysis	cutadapt-1.2.1, FASTX-Toolkit-0.0.13, Bowtie2-2.1.0, Tophat-2.0.14, Samtools-0.1.19, ImageJ-1.52, Vienna RNA package v2.0.5, statsmodels-0.12.2, bayes_opt-1.2.0, SciPy-1.5.2, Biopython-1.76, scikit-learn-0.21.3, cocor-1.1-3, aglip-3.12.0, GEOquery-2.48.0, Annotate-1.58.0, limma-3.36.5.

For manuscripts utilizing custom algorithms or software that are central to the research but not yet described in published literature, software must be made available to editors and reviewers. We strongly encourage code deposition in a community repository (e.g. GitHub). See the Nature Research guidelines for submitting code & software for further information.

Data

Policy information about availability of data

All manuscripts must include a data availability statement. This statement should provide the following information, where applicable:

- Accession codes, unique identifiers, or web links for publicly available datasets
- A list of figures that have associated raw data
- A description of any restrictions on data availability

The raw sequencing data, expression levels, and fold changes are deposited in GEO (accession number: GSE115646).
All the raw data are provided in the Source Data File.

Field-specific reporting

Please select the one below that is the best fit for your research. If you are not sure, read the appropriate sections before making your selection.

☒ Life sciences ☐ Behavioural & social sciences ☐ Ecological, evolutionary & environmental sciences

For a reference copy of the document with all sections, see nature.com/documents/nr-reporting-summary-flat.pdf

Life sciences study design

All studies must disclose on these points even when the disclosure is negative.

Sample size	No statistical methods were used to predetermine sample size. Sample size was determined based on experience and previous studies to reach statistical significance. All samples size details for the analyses carried out in this study are reported in the Figure legends and Methods.
Data exclusions	No data were excluded from the analysis.
Replication	For sequencing samples, multiple number of replicates (2 to 11) were generated to calculate averaged RPKM values with less noise. For experimental validations, a minimum of 3 three biological replicates were used and are indicated in the figure legends. Western blot results were repeated twice. All findings were reliably reproducible.
Randomization	Cells were cultured and the plates/wells were selected randomly to be treated with different conditions (transfection of different constructs or miRNAs, and mock treatment).
Blinding	The experimenters were not blinded for all studies. Blinding was not necessary because of the unbiased nature of the performed experiments. Appropriate positive and negative controls were used for each experiment.

Reporting for specific materials, systems and methods

We require information from authors about some types of materials, experimental systems and methods used in many studies. Here, indicate whether each material, system or method listed is relevant to your study. If you are not sure if a list item applies to your research, read the appropriate section before selecting a response.

Materials & experimental systems

n/a	Involved in the study
☐	☒ Antibodies
☐	☒ Eukaryotic cell lines
☒	☐ Palaeontology and archaeology
☒	☐ Animals and other organisms
☒	☐ Human research participants
☒	☐ Clinical data
☒	☐ Dual use research of concern

Methods

n/a	Involved in the study
☒	☐ ChIP-seq
☒	☐ Flow cytometry
☒	☐ MRI-based neuroimaging

Antibodies

Antibodies used	Primary antibodies used in this study are listed below:<br>anti-PCBP2, Rabbit poly-clonal, MBL-RN025P (1:2500)<br>anti-IGF2BP1, Rabbit poly-clonal, MBL-RN007P (1:5000)<br>anti-PABP1, Rabbit poly-clonal, Abcam-ab21060 (1:1000)<br>anti-LARP4, Rabbit poly-clonal, Bethyl Lab-A303-900A (1:1000)<br>anti-AGO2, Rabbit mono-clonal,Cell Signalling-2897S (1:1000)<br>anti-FLAG, Mouse mono-clonal, Sigma-F3165 (1:5000)<br>anti-GAPDH, Rabbit mono-clonal, Cell Signalling-2118S (1:2500)<br><br>Secondary antibodies used in this study are listed below:<br>Goat anti-rabbit IgG, BIO-RAD, 170-6515 (1:10000)<br>Goat anti-mouse IgG, BIO-RAD, 170-6516 (1:10000)
Validation	All primary and secondary antibodies used in this study were purchased from the companies as listed in the top section and have been validated by their manufacturers. The species and application for the primary antibodies used in this study are shown below.<br><br>-anti-PCBP2, Rabbit poly-clonal, MBL-RN025P<br>Species reactivity: Human, Mouse, Rat; Applications: WB, IP, RIP, eCLIP; cited in 9 papers shown in the website: https://www.mblbio.com/bio/g/dtl/A/index.html?pcd=RN025P#u-pub

-anti-IGF2BP1, Rabbit poly-clonal, MBL-RN007P
Species reactivity: Human, Mouse; Applications: WB, IP, IC, RIP, eCLIP, iCLIP; cited in 20 papers shown in the website: https://www.mblbio.com/bio/g/dtl/A/index.html?pcd=RN007P

-anti-PABP1, Rabbit poly-clonal, Abcam-ab21060
Species reactivity: Human, Mouse, Rat; Applications: ICC, WB; cited in 88 papers shown in the website: https://www.abcam.com/pabp-antibody-ab21060.html

-anti-LARP4, Rabbit poly-clonal, Bethyl Lab-A303-900A
Species reactivity: Human, Mouse; Applications: WB, IP; cited in 3 papers shown in the website: https://www.bethyl.com/product/A303-900A/LARP4+Antibody

-anti-AGO2, Rabbit mono-clonal,Cell Signalling-2897S
Species reactivity: Human, Mouse, Rat, Monkey; Applications: WB, IP; cited in 120 papers shown in the website: https://www.cellsignal.com/products/primary-antibodies/argonaute-2-c34c6-rabbit-mab/2897

-anti-FLAG, Mouse mono-clonal, Sigma-F3165
Species reactivity: All; Applications: WB, IP, IC, EIA; cited in 7 papers shown in the website: https://www.sigmaaldrich.com/KR/en/product/sigma/f3165

-anti-GAPDH, Rabbit mono-clonal, Cell Signalling-2118S
Species reactivity: Human, Mouse, Rat, Monkey, Bovine, Pig; Applications: WB, IP, IF, FC; cited in 4095 papers shown in the website: https://www.cellsignal.com/products/primary-antibodies/gapdh-14c10-rabbit-mab/2118

Eukaryotic cell lines

Policy information about cell lines

Cell line source(s)	HepG2 (HB-8065), HeLa (CCL-2), HCT116 (CCL-247), and HEK293T (CRL-3216) cell lines were purchased from ATCC company.
Authentication	All cell lines used in this study were authenticated by short tandem repeat (STR) profiling.
Mycoplasma contamination	All the cell lines used in the study have been tested negative from any mycoplasma contamination.
Commonly misidentified lines (See ICLAC register)	None of the cell lines used are listed in the database of commonly misidentified cell lines.
